# Evolutionary consequences of a large duplication event in *Trypanosoma brucei*: Chromosomes 4 and 8 are partial duplicons

**DOI:** 10.1186/1471-2164-8-432

**Published:** 2007-11-23

**Authors:** Andrew P Jackson

**Affiliations:** 1 Wellcome Trust Sanger Institute, Wellcome Trust Genome Campus, Hinxton, Cambridgeshire. CB10 1SA. UK

## Abstract

**Background:**

Gene order along the genome sequence of the human parasite *Trypanosoma brucei *provides evidence for a 0.5 Mb duplication, comprising the 3' regions of chromosomes 4 and 8. Here, the principal aim was to examine the contribution made by this duplication event to the *T. brucei *genome sequence, emphasising the consequences for gene content and the evolutionary change subsequently experienced by paralogous gene copies. The duplicated region may be browsed online at

**Results:**

Comparisons of trypanosomatid genomes demonstrated widespread gene loss from each duplicon, but also showed that 47% of duplicated genes were retained on both chromosomes as paralogous loci. Secreted and surface-expressed genes were over-represented among retained paralogs, reflecting a bias towards important factors at the host-parasite interface, and consistent with a dosage-balance hypothesis. Genetic divergence in both coding and regulatory regions of retained paralogs was bimodal, with a deficit in moderately divergent paralogs; in particular, non-coding sequences were either conserved or entirely remodelled. The conserved paralogs included examples of remarkable sequence conservation, but also considerable divergence of both coding and regulatory regions. Sequence divergence typically displayed strong negative selection; but several features, such as asymmetric evolutionary rates, positively-selected codons and other non-neutral substitutions, suggested that divergence of some paralogs was driven by functional change. The absence of orthologs to retained paralogs in *T. congolense *indicated that the duplication event was specific to *T. brucei*.

**Conclusion:**

The duplication of this chromosomal region doubled the dosage of many genes. Rather than creating 'more of the same', these results show that paralogs were structurally modified according to various evolutionary trajectories. The retention of paralogs, and subsequent elaboration of both their primary structures and regulatory regions, strongly suggests that this duplication was a seminal development, stimulating functional innovation and fundamentally altering the genetic repertoire of *T. brucei *relative to other trypanosomatids.

## Background

The African trypanosome, *Trypanosoma brucei*, causes sleeping sickness and substantial human morbidity across Africa. The recently completed genome sequences for *T. brucei *and two related protistan parasites, *T. cruzi *and *Leishmania major *[1-3], have provided a basis for understanding the biological and pathological differences among the Trypanosomatidae infecting humans. All trypanosomatid genomes share broad conservation of synteny, polycistronic transcription and a general absence of *cis*-introns (but isolated instances have been identified [2]). However, the number and size of chromosomes is known to vary between and within species, due to variation in repetitive, telomeric regions [4,5] and the infrequent, irregular genetic exchange between strains [6-8]. The *T. brucei *haplotype includes 11 megabase-sized chromosomes, as well as numerous mini-chromosomes [9]; other species have many more indicating that *T. brucei *has experienced a sequence of chromosomal fusions [10]. This study shows that the *T. brucei *genome has acquired a previously unreported duplication affecting chromosomes 4 and 8, which does not reflect temporary karyotypic fluctuation. The report begins by documenting this partial chromosomal duplication to identify paralogous gene copies, and then a quantitative analysis of paralogous sequences examines the potential for evolutionary innovation and the importance of the duplication for the genomic repertoire in *T. brucei*.

The architecture of genome sequences has shown that duplication is a frequent and important process in genome evolution [11,12]. It occurs on every scale within the genome: mistakes during DNA synthesis cause tandem duplication of individual genes, and segmental duplication often results from the transposition of mobile elements; for instance, the transposition of an *Alu *element caused the duplication of the human BRCA1 region [13]. Duplication of whole chromosomes, or chromosome-sized blocks, can result from mistakes during cell division, (i.e., non-disjunction). This can also occur after whole genome duplication (WGD) when a polyploid genome decays through selective gene loss. The relative importance of these processes seems to vary by gene function and taxon; WGD has been widely reported, most notably among plants [14-17] and yeasts [18-21], and may have been responsible for major evolutionary transitions in chordates [22-24]. Certainly, the prevalence and importance of duplication in genomic evolution has only recently become clear [11] and is among the major insights delivered by whole genome sequencing. These observations have helped to revive the argument of Ohno [25], eclipsed in the pre-genomic era by the focus on sequence evolution, that gene duplication is the principal source of evolutionary novelty [reviewed in [12]], faster and more consequential than nucleotide substitution.

The fate of gene duplicates seems to be multifarious and subject to various factors. Since duplications of any kind disrupt systems at, or near, optimality, one should assume that most duplications are selected against. Indeed, most loci created after large duplication events are subsequently deleted [12,18], resulting in 'diploidisation' in *Arabidopsis thaliana *for example [22,26]. However, gene loss is neither complete nor random, and may show similar trends across taxa [27]; in teleost fishes, genes associated with signalling and gene regulation were enriched following gene loss [28], transcription factors were over-represented in rice [29], while *A. thaliana *preferentially retained signal transduction loci [26]. Loci with a high level of proteomic connectivity were also selectively retained in *A. thaliana *following WGD [26]. Ohno's original model [25] intuitively suggested that duplication facilitated novel functions (neofunctionalisation) through the relaxation of purifying selection due to redundancy after duplication. The importance of rapid, structural evolution to functional innovation has been inferred from the widespread asymmetry of evolutionary change among paralogous genes and regions [19,26,30-32], the acceleration of evolutionary rate among paralogs [33,34] and positive selection of duplicated genes [35,36].

In contrast to neofunctionalisation, functional change might result from a segregation of the original gene function between duplicates (subfunctionalisation), due to degenerate mutations in each, and producing copies with distinct specificities [37-40]. The duplication-degeneration-complementation model [38] refined this concept, stating that complementary mutations in regulatory regions were responsible for partitioning functionality. Many examples of duplicates performing generic functions, but with specific spatial or temporal expression profiles, are known; *myb*-homologs in maize (*Zea mays*) are tandem duplicates and are expressed in distinct flower tissues due to divergence in their 3' regulatory regions [41]. In pufferfish (*Takifugu rubripes*), two copies of a *Hox *gene (*Hoxa2*) formed after WGD are expressed in distinct regions of the hindbrain, whereas their common ortholog in tetrapods is expressed throughout; tissue specificity evolved through changes in *cis *regulatory modules [42]. Along with loss of function (pseudogenisation, or nonfunctionalisation), a gallery of potential fates has been formulated. What is clear is that sensible changes in structure are correlated with changes in function; these may affect coding sequences or regulatory modules, and the precise outcome of duplication likely reflects both selective pressures (adaptation) and historical constraints, that is, the function, indispensability and connectivity of the original gene [43]. Duplicates may evolve through structural divergence or rapid changes in expression profile, but duplication always creates evolutionary opportunities, some of which may lead to novelty.

This study provides the first account of a large duplication event in *T. brucei*, which, if its consequences are similar to those in other organisms, may provide, or have provided, the raw material for evolutionary innovation and the expansion of gene families. Previously, studies of tandem gene arrays had documented gene duplication in trypanosomatids, for example, the phosphoglycerate kinase gene array [44] and hexose transporters [45], where gene duplication combined with gene conversion to create novel sequence types; this phenomenon is now known to be widespread [46]. Elaboration of important trypanosomatid gene families, such as amastin surface antigens in *Leishmania *spp. [47] and VSGs in *T. brucei *[48] are also the consequences of gene duplication. However, the impact of this chromosomal duplication has been much greater and, by examining the evolutionary changes that have subsequently affected each duplicon, this study sought to establish its contribution to the *T. brucei *genome. There were four specific aims: i) to document gene losses and gains since the duplication event; ii) to describe patterns of both coding and non-coding sequence divergence between paralogous gene pairs ; iii) to assess evidence for disparity in evolutionary rate during divergence using relative rates tests; iv) to assess the role of non-neutral substitutions in deriving new functions.

## Results

### (a) Partial chromosomal duplication: gene content and order

**Figure 1:**
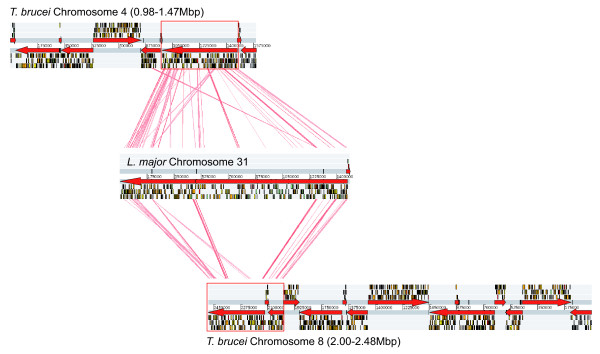
ACT comparison of whole Chromosomes 4 and 8 in *T. brucei *and Chromosome 31 in *L. major*. Chromosomes are presented as in GeneDB, with both forward and reverse strands and loci represented as coloured rectangles; grey arrows indicate the direction of transcription along polycistronic regions. Red rectangles denote the boundaries of the duplicated region on each chromosome; significant tBLASTx matches between homologous genes are linked by red coloured lines. Note that, while indicative, not all sequence affinity between chromosomes was detected in this tBLASTx search.

Comparison of gene order along Chromosomes 4 and 8 of *T. brucei *with homologous regions in *T. cruzi *and *L. major *demonstrated widespread colinearity within and between species. Figure 1 shows tBLASTx analyses between Chromosomes 4 and 8 and Chromosome 31 in *L. major*, visualised by the Artemis Comparison Tool (ACT). The blast hits between chromosomes 4 and 8 are given in greater detail in Additional data file 1. On Chromosome 8 (0.98–1.47 Mb), the duplicon begins with a ser/thr-protein kinase NrkAgene (**1**: Tb927.8.6930; paralogous gene pairs are numbered **1 **to **74 **and referred to by their GeneDB identifier tags), which is preceded upstream by a strand-switch region, and ends at the chromosomal terminus with a receptor-type adenylate cyclase (**74**: Tb927.8.8360). On Chromosome 4 (2–2.48 Mb), a paralog of the ser/thr-protein kinase NrkAgene is found near the chromosomal terminus (**1**: Tb927.4.5390) and is followed downstream by a strand-switch region and several genes typical of *T. brucei *sub-telomeric regions. Synteny with Chromosome 8 is conserved upstream, culminating in several genes that include a receptor-type adenylate cyclase (**74**: Tb927.4.3860). This is preceded upstream by 10 loci that were not conserved on Chromosome 4, and then a strand-switch region. Hence, each duplicon was bound by strand-switch regions and approximately 0.5 Mb in size, and gene order between the two regions was colinear and anti-parallel.

The location of homologs of the duplicated genes in *T. cruzi *and *L. major *genome sequences showed that gene order was largely conserved in these species. This served two purposes; first, it showed that both duplicons corresponded to the entirety of Chromosome 31 in *L. major*, although, as Figure 1 shows, this chromosome is larger and contains other genes besides those retained in *T. brucei*. Complete chromosomal structure is not established in *T. cruzi*. And second, it enabled the ancestral gene order of the pre-duplication chromosome to be inferred, through the classification of duplicated genes as *shared *(i.e. present on both duplicons and in an outgroup), *lost *(i.e. present on one duplicon and in an outgroup) or *gained *(not present in an outgroup). 74 loci were shared by both duplicons and other species. 57 genes on Chromosome 4 were absent from Chromosome 8 but present in other species, indicating that they were lost. Similarly, 27 genes on Chromosome 8 and present in other species were absent from Chromosome 4. Therefore, 47% of all duplicated loci were retained as paralogs on both duplicons. Furthermore, 7 and 18 loci were present on Chromosomes 4 and 8 respectively, but absent from other species, suggesting that they were independently gained post-duplication. A detailed and interactive figure showing the colinear gene order of the duplicons, and with links to the *T. brucei *genome sequence, is available from the GeneDB website [49], (also included here as Additional data file 1). Shared and lost genes appeared to differ in the presence of transmembrane helices (TMH) and putative signal peptides. A two-sample t-test assuming heteroscedasticity confirmed that conserved paralogs included significantly more TMH (μ = 1.45, df = 102, p = 0.011) and signal peptides (μ = 0.2, df = 134, p = 0.033) than singleton genes.

It was concluded from comparison of *T. brucei *and *T. congolense *genome sequences that the segmental duplication is restricted to *T. brucei*. It was initially observed that the preliminary assembly for chromosomes 4 and 8 in *T. congolense *did include homoeologous regions to the *T. brucei *duplicons. The putative duplicons in *T. congolense *showed conserved synteny and numerous retained paralogs, as in *T. brucei*. However, comparison of sequence divergence between paralogs showed that the preliminary *T. congolense *genome sequence did not contain an ortholog for each paralog seen in *T. brucei*, as expected if the duplication had occurred prior to speciation. Of 42 instances where a locus had been duplicated and retained on both duplicons, by both species, the *T. congolense *'paralogs' were identical in nucleotide sequence in every case; furthermore, the intergenic sequences of 'paralogous' regions were also identical. Further examination of the sequence reads for *T. congolense *homologs identified putative alleles, but nothing to suggest the presence of orthologs to both *T. brucei *duplicates. It is implausible that while *T. brucei *paralogs have diverged considerably in most cases, and intergenic regions have little or no affinity, the corresponding regions in *T. congolense *should have remained entirely unchanged over the same time-span. With the completion of the *T. congolense *genome, it will hopefully become clear why preliminary assemblies reproduced the structure of the *T. brucei *genome sequence; but *T. congolense *certainly does not display the evolutionary dynamics seen in *T. brucei *and does not share in the effects of the duplication event (in terms of the derivation of novel genes).

### (b) Sequence divergence of conserved paralogs

**Figure 2:**
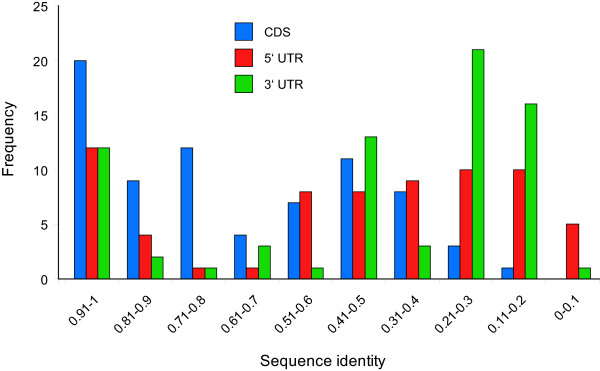
Frequency distribution of sequence identity between paralogs retained on both duplicons. Nucleotide sequence identity is recorded for coding regions and each UTR.

The remaining analyses dealt with the consequences of segmental duplication for the divergence of conserved paralogs present on both duplicons. There was considerable variation in the sequence identity between paralogs, as shown in Additional data file 1 and recorded in Additional data file 2. There were instances of extreme conservation between paralogs, for example, a myosin heavy chain kinase A showing 98% identity (**26**: Tb927.4.4970 and Tb927.8.7450), and of extreme divergence, for example, a monoglyceride lipase showing 39% identity (**53**: Tb927.4.4360 and Tb927.8.8020). Other paralogous, hypothetical genes showed as little as 5% identity (e.g., **7**: Tb927.4.5330 and Tb927.8.7060). Figure 2 shows a bimodal frequency distribution of nucleotide sequence identity for all shared paralogs. Coding sequences either changed little, retaining 70–100% identity, or diverged to around 40% identity; but there were low numbers of CDS with identity at 50–60%, or less than 30%.

**Figure 3:**
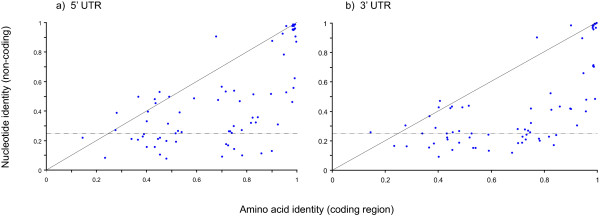
Sequence divergence between paralogs. Values for coding regions are correlated with 5' UTR (a) and 3'UTR (b) regions. Horizontal dashed lines denote the 25% nucleotide identity expected between two unrelated sequences.

Sequence divergence among NCS was generally greater and bimodality was more pronounced. There were paralogs with almost identical 5' and 3' untranscribed regions (UTR), for instance, paralogous RNA polymerase IIA large subunits (**22**: Tb927.4.5020 and Tb927.8.7400) had identical 3' UTRs over 400 bp; but highly divergent, indeed unalignable, UTRs were a more typical observation. Figure 2 identifies many NCS with less than 25% identity, which is no greater than expected by chance. These cases are largely an expression of comparisons between unaligned sequences; however, in some instances a part of the CDS or NCS aligned well but constituted only a minor fraction of the whole alignment, producing a value below 0.25. This explains how paralogy was established between sequences with less than 0.25 identity. The bimodality of sequence divergence is further illustrated in Figure 3 where coding and non-coding identities for each paralogous gene pair are correlated. Both panels A and B, though especially the 3' UTR comparison, clearly show gene pairs consistent with a 1:1 relationship, including both conserved and divergent cases. However, in both cases there was a deficit of gene pairs falling in the 0.6–0.8 range and a surfeit of those falling into the bottom-right quadrant of the graph. These cases have CDS identities between 0.6 and 0.8, but with much lower values for NCS. Once again, many NCS values fell below 0.25, reflecting those sequences that were unaligned. In summary, despite being formed at the same time, divergence of duplicated coding and non-coding sequences varied widely along the duplicons. The bimodality of divergence values indicated that fewer duplicate sequences had diverged moderately than expected; rather duplicates had remained largely unchanged or become very different.

### (c) Evolutionary rate asymmetry

Relative rates tests were used to analyse paralogous CDSs for significant asymmetry in the rate of evolution post-duplication; all results are shown in Additional data file 3. Canonical relative rates tests identified 12 cases where one paralog had evolved significantly faster; these are listed in Table 1, and indicated by blue arrows in Additional data file 1. Among these cases there were two gene pairs, 3-methylcrotonyl-CoA carboxylase (**5**: Tb927.4.5350 and Tb927.8.6970) and dihydrolipoamide dehydrogenase (**20**: Tb927.4.5050 and Tb927.8.7380), where the rate asymmetry coincided with apparent psuedogenisation of one paralog. Elsewhere, monoglyceride lipase, noted above as having exceptional sequence divergence also displayed rate asymmetry (in favour of the Chromosome 8 copy), as did paralogous tandem gene arrays of glycosyltransferase (**57**: Tb927.4.4290 and Tb927.8.8090) and amino acid transporter genes (**69**: Tb927.4.4020 and Tb927.8.8220). The largest asymmetry in evolutionary rate occurred between paralogs of a mitotic centromer-associated kinesin (**73**: Tb927.4.3910 and Tb927.8.8350); the rate of non-synonymous substitutions per non-synonymous site for the Chromosome 4 lineage was 0.4386, compared with 0.6062 for the Chromosome 8 lineage (p = 1.00 × 10^-7^). The Bayesian relative rates test was more conservative, identifying 4 cases of significant asymmetry that are detailed in Table 2; each of these corroborated a significant result obtained using the canonical test. A complete account of the Bayesian relative rates tests is given in Additional data file 4.

**Table 1:** Shared, paralogous loci showing significant asymmetry in the rate of non-synonymous substitutions per site since duplication, as determined by the canonical relative rates test.

**Locus**	**Identifer**		**Description**	**CDS identity**	**Interspecific CDS**		**5' UTR:**		**3' UTR:**		**RRT:**						
	Chr4	Chr8			Identity:		Identity	Length (bp)	Identity	Length (bp)	# Sites	*D*_*n*_		Δ*D*_*n*_	SD	ratio	P
					Chr4	Chr8						Chr4	Chr8				
5	Tb927.4.5350	Tb927.8.6970	3-methylcrotonyl-CoA carboxylase	0.983	0.661	0.652	0.976	42	0.984	327	1361.5	0.253	0.241	-0.012	0.004	-2.797	0.005
9	Tb927.4.5310	Tb927.8.7110	S/T-protein kinase A	0.845	0.684	0.714	0.357	8	0.239	~250	887.1	0.141	0.184	0.043	0.011	3.862	0.000
20	Tb927.4.5050	Tb927.8.7380	dihydrolipoamide dehydrogenase	0.924	0.621	0.647	0.308	0	0.303	18	1085.0	0.268	0.255	-0.013	0.005	-2.647	0.008
43	Tb927.4.4550	Tb927.8.7780	Tb927.8.7760	0.516	0.453	0.366	0.247	0	0.436	0	1723.4	0.439	0.606	-0.168	0.023	-7.302	0.000
46	Tb927.4.4520	Tb927.8.7820	Tb927.8.7800	0.407	0.218*	0.242*	0.396	80	0.47	500?	1881.0	0.886	0.764	0.122	0.035	3.500	0.000
51	Tb927.4.4350	Tb927.8.8030	Tb927.8.7950	0.433	0.239	0.261	0.156	0	0.25	0	3308.0	0.939	0.863	0.076	0.028	2.729	0.006
54	Tb927.4.4380	Tb927.8.7980	monoglyceride lipase	0.783	0.616	0.553	0.098	0	0.199	0	610.9	0.300	0.343	-0.043	0.018	-2.346	0.019
58	Tb927.4.4240	Tb927.8.8070	glycosyltransferase	0.519	0.438	0.416	0.192	0	0.222	0	753.3	0.497	0.565	-0.068	0.034	-1.975	0.048
59	Tb927.4.4220	Tb927.8.8140	Tb927.8.8070/8110	0.145	0.13*	0.098*	0.219	0	0.258	0	151.5	0.547	0.930	-0.384	0.117	-3.270	0.001
70	Tb927.4.3970	Tb927.8.8320	amino acid transporter	0.734	-	-	0.261	0	0.241	0	944.1	0.220	0.182	0.037	0.015	2.506	0.012
73	Tb927.4.3910	Tb927.8.8350	Tb927.4.3920 (TMH)	0.727	0.609	0.559	0.166	0	0.276	0	564.4	0.265	0.360	-0.095	0.023	-4.127	0.000
74	Tb927.4.3880	Tb927.8.8360	mitotic centromer-associated kinesin	0.686	0.43	0.432	0.473	190	-	-	1339.9	0.497	0.536	-0.039	0.018	-2.141	0.032

Locus number refers to paralogous loci retained on both duplicons, as described in Additional files 1 and 2.

An asterisk * denotes the use of *T. vivax *as the outgroup comparison, in place of *T. cruzi*.

**Table 2:** Shared, paralogous loci showing significant asymmetry in total substitution rate since duplication (P < 0.05), as determined by the Bayesian relative rates test.

Locus	Identifier		Chromosome 4:				Chromosome 8:			
	Chr4	Chr8	Average	SD	Range		Average	SD	Range	
42	Tb927.4.4570	Tb927.8.7760	0.4483	0.0220	0.404	0.492	**0.5771**	0.0265	**0.524**	**0.630**
45	Tb927.4.4530	Tb927.8.7800	**0.4914**	0.0284	**0.435**	**0.548**	0.3339	0.0240	0.286	0.382
50	Tb927.4.4400	Tb927.8.7950	**0.6422**	0.0269	**0.588**	**0.696**	0.5099	0.0232	0.464	0.556
58	Tb927.4.4240	Tb927.8.8070	0.4534	0.0774	0.299	0.608	**0.8414**	0.1162	**0.609**	**1.074**

Locus number refers to Additional file 1.

Values in bold denote the paralogs with the faster evolutionary rate.

### (d) Non-neutral evolution

One factor capable of causing differential evolutionary divergence between paralogs is natural selection. For all genes along the duplicons, the action of selection on individual codons was examined using SLAC, FEL and REL algorithms to calculate *ω*. The *T. congolense *homolog was used as an outgroup comparison in these tests. Additional file 5 shows the global *ω *values for all loci, none of which suggested net positive selection (i.e., *ω *> 1). Most singleton loci and paralogous genes were under negative, purifying selection when compared to their *T. congolense *homologs (i.e., *ω *<< 1), which was reflected in a left-skewed frequency distribution of global *ω *and an average value of 0.219 (± SD 0.099). Those paralogs showing extreme deficits in non-synonymous substitutions (i.e., *ω *< 0.1) included an RNA polymerase IIA large subunit that was generally well conserved (**22**: Tb927.4.5020 and Tb927.8.7400), an ubiquinol-cytochrome C reductase (**25**: Tb927.4.4990 and Tb927.8.7430) and a translocating pyrophosphatase (**51**: Tb927.4.4380 and Tb927.8.7980), despite this being generally quite divergent. Although purifying selection was ubiquitous, FEL and REL tests detected 12 and 41 positively-selected codons respectively among both singleton loci and retained paralogs. However, the incidence of positive selection involving paralogous genes (and their outgroup) was significantly higher than for singletons and their outgroups, when combining the two tests (p = 0.0125, Fisher's exact test) or analysing them separately (FEL, p = 0.0059; REL, p = 0.051). Including an outgroup in these tests meant that some cases of positive selection could derive from the interspecific comparison, rather than the duplication event. Hence, when the outgroup was excluded, positive selection was detected in 21 duplicate loci; Table 3 describes the results for 16 loci that included positively-selected loci both with and without an outgroup comparison (these are also marked with red arrows in Additional file 1).

**Table 3:** Retained paralogs with codons showing evidence for positive or negative selection.

Locus	Identifier Chr4	Chr8	Selection analysis: With outgroup					Without outgroup		
			Method	Global ω	+	-	+ codons	- codons	+ codons	- codons
6	Tb927.4.5340	Tb927.8.6980	SLAC	0.382	0.369	0.462	0	0		
			FEL				0	54		
			REL				2	65	67	0
8	Tb927.4.5320	Tb927.8.7090	SLAC	0.333	0.325	0.418	0	0		
			FEL				0	84		
			REL				5	340	7	6
10	Tb927.4.5300	Tb927.8.7140	SLAC	0.289	0.271	0.364	0	1		
			FEL				1	44		
			REL				15	192	20	7
12	Tb927.4.5220	Tb927.8.7190	SLAC	0.315	0.302	0.415	0	0		
			FEL				0	31		
			REL				6	77	19	7
14	Tb927.4.5180	Tb927.8.7220	SLAC	0.201	0.192	0.266	0	0		
			FEL				0	73		
			REL				4	135	2	49
21	Tb927.4.5030	Tb927.8.7390	SLAC	0.072	0.062	0.112	0	0		
			FEL				0	65		
			REL				5	286	3	24
27	Tb927.4.4960	Tb927.8.7460	SLAC	0.188	0.176	0.246	0	0		
			FEL				0	76		
			REL				7	289	49	0
31	Tb927.4.4920	Tb927.8.7500	SLAC	0.413	0.363	0.508	0	0		
			FEL				0	13		
			REL				6	63	15	0
40	Tb927.4.4730	Tb927.8.7740	SLAC	0.148	0.138	0.202	0	1		
			FEL				0	45		
			REL				4	201	40	0
43	Tb927.4.4550	Tb927.8.7780	SLAC	0.199	0.193	0.261	0	1		
			FEL				0	158		
			REL				11	17	5	7
46	Tb927.4.4520	Tb927.8.7820	SLAC	0.342	0.33	0.426	0	0		
			FEL				1	57		
			REL				1	73	6	0
49	Tb927.4.4470	Tb927.8.7860	SLAC	0.256	0.253	0.335	0	2		
			FEL				0	214		
			REL				2	187	20	0
50	Tb927.4.4400	Tb927.8.7950	SLAC	0.369	0.365	0.447	0	10		
			FEL				4	191		
			REL				0	121	2	0
52	Tb927.4.4370	Tb927.8.8000	SLAC	0.216	0.207	0.28	0	0		
			FEL				0	91		
			REL				49	68	8	0
62	Tb927.4.4160	Tb927.8.8170	SLAC	0.282	0.273	0.345	0	1		
			FEL				1	94		
			REL				3	181	5	0
71	Tb927.4.3950	Tb927.8.8330	SLAC	0.249	0.242	0.318	0	0		
			FEL				1	115		
			REL				14	458	27	41

Beyond the ratio of amino acid replacements to silent substitutions, other patterns of sequence divergence could reflect non-neutral evolution. Paralogous sequences were scored for the ratio of 'invariable' to 'variable' mutations at non-synonymous and then synonymous sites. Significant disparity between these ratios was an indication of non-neutral evolution and was detected in 15 cases at the p = 0.01 level; these are shown in Table 4 and as green arrows on Additional data file 1. Details for all loci are shown in Additional data file 6. These comprised the tail in an over-dispersed distribution of G statistics, meaning that most cases showed negligible difference between substitution patterns at the distinct sites. Where significant disparity was observed, this was generally due to an excess of RI mutations, and there were four cases in particular where the number of invariable mutations outnumbered variable mutations at non-synonymous sites; these concerned three pairs of paralogous hypothetical genes: **6 **(Tb927.4.5340 and Tb927.8.6980), **2 **(Tb927.4.5380 and Tb927.8.6940), and **24 **(Tb927.4.5000 and Tb927.8.7420); as well as paralogs of a single-copy amino acid transporter (**40**: Tb927.4.4730 and Tb927.8.7740).

**Table 4:** Shared, paralogous loci showing significant disparity (p < 0.005) between the ratio of invariable to variable mutations at synonymous (S) and non-synonymous sites (R) respectively, as determined by G test.

Locus	Description	Mutation type:				G	Ratio_R_	Ratio_S_	Ratio_RS_
		RI	RV	SI	SV				
2	Tb927.4.5380 (alcohol dehydrogenase-like)	436	10	310	21	8.155	43.60	14.76	2.954
6	Tb927.4.5340	356	284	147	191	13.03	1.254	0.770	1.629
8	Tb927.4.5320	197	384	105	314	9.131	0.513	0.334	1.534
24	Tb927.8.7420	256	5	210	15	6.937	51.20	14.00	3.657
29	Tb927.8.7480	135	484	53	317	8.674	0.279	0.167	1.668
35	Tb927.8.7580 (TMH/SP)	201	539	75	400	22.05	0.373	0.188	1.989
36	amino acid transporter	132	279	89	379	19.92	0.473	0.235	2.015
37	amino acid transporter	54	191	41	287	9.081	0.283	0.143	1.979
41	amino acid transporter	139	60	83	77	12.09	2.317	1.078	2.149
49	Tb927.8.7850	210	598	84	429	17.25	0.351	0.196	1.793
50	adenylate cyclase GRESAG 4	73	502	98	414	8.458	0.145	0.237	0.614
51	Tb927.8.7950	430	1671	178	905	7.649	0.257	0.197	1.308
58	UDP-GlcNAc-dependent glycosyltransferase	67	318	24	247	10.12	0.211	0.097	2.168
62	Tb927.4.4180	234	775	83	475	15.94	0.302	0.175	1.728
68	Tb927.8.8270	263	748	97	485	19.05	0.352	0.200	1.758

### (e) Taxonomic distribution PCR assay

Three locations were selected where a shared paralog on both chromosomes was followed downstream by different, single-copy genes on each duplicon. For each location, a genome containing both duplicons should yield two distinct PCR products. The presence of both duplicons in other *T. brucei *strains would therefore be demonstrated by amplification of all six PCR products of the correct size. Additional data file 7 shows that this was observed in the subspecies to which the original genome sequence belongs (*T. b. brucei*), but also in *T. b. gambiense*, *T. b. rhodiense *and *T. evansi*, confirming that the duplication was common to all members of the *T. brucei *clade.

## Discussion

The duplication of a chromosome-sized region of the *T. brucei *genome was identified, based on the colinear gene order along 0.5 MB regions of chromosomes 4 and 8. Comparisons with homoeologous regions in other trypanosomatids confirmed that the duplicated region corresponded to the entire chromosome 31 in *Leishmania *spp. and was unique to *T. brucei*. In addressing the aims of this study, it has been shown that although substantial gene loss occurred after duplication, 47% of all duplicated loci had been retained as conserved paralogs. The functions of retained compared with deleted genes suggested that gene loss was selective. Divergence of conserved paralogs was bimodal, particularly in the case of NCSs; UTRs either remained highly conserved or were radically remodelled. Sequence divergence was also characterised by ubiquitous purifying selection, frequent rate asymmetry between paralogs and occasional positive selection, which nonetheless occurred significantly more often among duplicates than single-copy genes. So taken together, the patterns of observed structural change suggested that at least some conserved paralogs were functionally innovative.

### (a) Post-duplication effects on gene content

Duplication events of this kind have not previously been recorded in trypanosomatid genomes. Karyotypic fluctuations appear to be reasonably frequent among trypanosomatids; in *T. brucei *the infrequent nature of reductive cell division produces triploid hybrid strains [50,51]; the irregularity of genetic exchange in these organisms also seems to cause widespread variation in ploidy in *T. cruzi *[7]. Among *Trypanosoma *spp. and *Leishmania *spp. fluctuations in repetitive, telomeric regions causes substantial variation in chromosome size [4,5]. However, the effect of these events seems to be restricted to karyotype, and has not had permanent effects on genetic complement. The duplication event recorded here differs in nature because its effects on genetic complement go beyond spatio-temporal fluctuations in copy number; the duplicons evolved through deletion of many genes, gain of a few others, and widespread divergence of retained paralogs, to create a novel and permanent addition to the *T. brucei *genome. Such expansions in genetic complement through large duplication events have emerged as primary evolutionary catalysts from various taxa; both yeasts [18-20] and angiosperms [14-17] are known to be palaeopolyploids, while the possibility of successive whole-genome duplications in vertebrates continues to be debated [24,51-55].

Comparisons of completed genomes from these organisms are illuminating the mechanisms of large duplication events. Koszul et al. (2003) [56] examined reversion to wild-type in *Saccharomyces cerevisiae *after enforcing a growth defect; the majority of revertant strains resulted from spontaneous duplication events, ranging in size between 41 and 655 kb. This and other observations [57] suggest that large duplication events result from damage incurred during DNA replication and its subsequent repair. The locations of such breakpoints in yeast also support the view that damage occurs at specific points of weakness, for instance termination sites, repetitive regions and those containing mobile elements [58,59]. Similarly, in *T. brucei*, the upstream junction of the chromosome 4 duplicon comprised a 40 kb region with almost no open reading frames but several mini-satellite loci and both DIRE and RIME mobile elements.

Large duplication events make immediate additions to genomic repertoire, many of which will prove lasting. However, the conclusion from several model organisms is that such events are typically followed by substantial gene loss [11-18], often resulting in 'diploidisation' and the restoration of original gene number [22]. Unusually, almost half of duplicated loci recorded here are retained as paralogs; this contrasts with 28.6% and just ~20% following whole-genome duplications in *A. thaliana *[60] and *Oryza sativa *[29] respectively, and perhaps 10% in yeast [61]. Such recognised WGD events are shared across species or genera, and are undoubtedly ancient. By contrast, the present case is apparently restricted to *T. brucei *and is certainly absent from other principal trypanosome clades. Its cladistic distribution suggests that is a relatively recent event, which may explain the large fraction of genes retained in comparison with other large duplication events. The fate of gene duplicates is complex and depends both on taxon [27] and function, incorporating protein complexity [62], dose sensitivity and 'connectivity' [60,63,64]. Some observations suggest that genes encoding simpler products, with fewer interactions with regulators or targets around the cell (i.e., lower 'connectivity'), are retained in duplicate more often. Hence, in yeast complex proteins are retained less often as the number of subunits increases [65], subunits of heterodimers are less duplicable than those in homodimers [62] and there was a negative relationship between 'connectivity' and retention after duplication [66]. The 'balance' hypothesis explains such biases in terms of dose sensitivity [63]; effective gene expression depends on a dynamic equilibrium of regulatory factors, which is perturbed by unilateral duplications of regulatory genes or individual components of larger assemblages. Hence, genes that are more peripheral to the regulatory environment and dose-insensitive, such as surface-expressed genes, may be retained more often [66].

However, most analyses identifying selective gene loss report enrichment of highly connected and expressed genes, integral to cell function, for instance those associated with regulation, signal transduction, transcriptional control and protein-protein interactions [26,27]; this can apparently result in co-localisation of regulator and target loci over time [60,67]. Yet, these studies have addressed WGD events and the dosage balance hypothesis accommodates them if gene loss is considered scale-dependent [68]. Dosage balance can be preserved either by retaining all components of a regulatory network, where a duplication is large enough to include a gene plus all its interacting loci, or by deleting them, where the event has been smaller. In *T. brucei*, retained paralogs were enriched with TMH and signal peptides, showing that gene loss was selective after duplication, resulting in preferential retention of surface expressed genes, (e.g., amino acid transporters, adenylate cyclases, glycosyltransferases). Although the large number of uncharacterised genes limits our ability to scrutinise gene loss by function, metabolic enzymes such as metallopeptidases, components of the electron transfer chain, and various loci associated with the RNA synthesis and modification featured prominently among deleted duplicates. This is consistent with the preservation of dosage balance through deletion of loci with high connectivity, and retention of dose-insensitive proteins on the cell surface. Indeed, the process of removing dose-sensitive genes may be continuing through pseudogenisation of duplicates such as a dihydrolipoamide dehydrogenase (**20**: Tb927.8.7380). Prior to duplication, this locus was duplicated in tandem; after duplication, one copy was lost from chromosome 8 and another is currently being deleted from chromosome 4, thereby restoring the original copy number.

### (b) Post-duplication effects on gene sequence

For those gene duplicates that are preserved, duplication marks the beginning of genetic divergence. Paralogs will diverge over time, unless gene conversion homogenises their sequences at a much faster rate [46]. The speed and magnitude of evolutionary change depends on the selective environment following duplication, the nature of which is likely to vary by case. The role of adaptive evolution in neofunctionalisation and subfunctionalisation models differs, but setting positive selection aside, the common theoretical expectations are that evolutionary rate should accelerate in one or both duplicates as functional change accumulates. Regulatory regions should also be remodelled, either to effect functional change (i.e., under a DDC model) or to preserve it. In this case, most features of sequence divergence – the prevalence of negative selection, rate asymmetry, and excessive numbers of non-synonymous substitutions – indicate that post-duplication sequence change has been functional. At a basic level, patterns of coding and non-coding sequence divergence displayed a deficit in moderate divergence, indicating that regulatory regions in particular remained conserved or were entirely remodelled. Therefore, given that paralogs could be conservative or innovative with respect to both CDSs and NCSs, it follows that four types of dynamics were observed, examples of which are listed in Table 5.

**Table 5:** Sequence divergence of shared, paralogous loci, displaying four different evolutionary dynamics.

**Locus**	**Identifier**		**Annotation**	**Sequence identity:**	
	Chr4	Chr8		CDS	3'UTR
**Conserved CDS/Conserved NCS**					
1	Tb927.4.5390	Tb927.8.6930	serine/threonine-protein kinase NrkA	0.983	0.968
2	Tb927.4.5380	Tb927.8.6940	quinonprotein alcohol dehydrogenase-like	0.993^a^	0.995
3	Tb927.4.5370	Tb927.8.6950	dynein light chain 2B	0.994	0.994
4	Tb927.4.5360	Tb927.8.6960	TMH/SP	0.987	0.984
5	Tb927.4.5350	Tb927.8.6970	3-methylcrotonyl-CoA carboxylase	0.983^b^	0.984
22	Tb927.4.5020	Tb927.8.7400	RNA polymerase IIA largest subunit	0.999	1
24	Tb927.4.5000	Tb927.8.7420	C2 calcium/lipid-binding region, CaLB	0.995^a^	0.966
30	Tb927.4.4930	Tb927.8.7490		0.987	0.956
64	Tb927.4.4150	Tb927.8.8180		0.991	0.995
65	Tb927.4.4140	Tb927.8.8190		0.986	0.981
67	Tb927.4.4120	Tb927.8.8210		0.984	0.982
					
**Divergent CDS/Divergent NCS**					
7	Tb927.4.5330	Tb927.8.7060	EGF/Laminin domain	0.235	0.165
10	Tb927.4.5240	Tb927.8.7140	UDP-GlcNAc-dependent glycosyltransferase	0.455	0.258
11	Tb927.4.5230	Tb927.8.7180		0.385	0.181
12	Tb927.4.5220	Tb927.8.7190	SP	0.402	0.09
18	Tb927.4.5120	Tb927.8.7260	kinetoplast-associated protein	0.368	0.365
29	Tb927.4.4940	Tb927.8.7480	phosphopantetheine attachment site	0.491^a^	0.427
33	Tb927.4.4900	Tb927.8.7550		0.282	0.16
34	Tb927.4.4890	Tb927.8.7560	TMH	0.454	0.137
35	Tb927.4.4880	Tb927.8.7580	TMH/SP, Zn-finger protein	0.48^a^	0.264
38	Tb927.4.4810	Tb927.8.7710	TMH	0.402	0.425
39	Tb927.4.4790	Tb927.8.7720	TMH/SP	0.391	0.288
42	Tb927.4.4580	Tb927.8.7750	protein kinase	0.446	0.422
46	Tb927.4.4530	Tb927.8.7800	SPla/RYanodine receptor SPRY	0.407^b^	0.47
47	Tb927.4.4520	Tb927.8.7820	cold-shock protein, DNA-binding	0.434	0.222
48	Tb927.4.4500	Tb927.8.7830		0.34	0.25
51	Tb927.4.4400	Tb927.8.7950	leucine rich repeat	0.433^ab^	0.25
57	Tb927.4.4310	Tb927.8.8050	spectrin repeat	0.366	0.152
59	Tb927.4.4240	Tb927.8.8070	Zinc finger, C2H2-type	0.145	0.258
60	Tb927.4.4220	Tb927.8.8140	small GTP-binding rab protein	0.452	0.433
62	Tb927.4.4180	Tb927.8.8160		0.48^a^	0.186
69	Tb927.4.4040	Tb927.8.8280		0.436	0.209
71	Tb927.4.3970	Tb927.8.8320		0.277	0.302
					
**Conserved CDS/Divergent NCS**					
9	Tb927.4.5310	Tb927.8.7110	serine/threonine-protein kinase A	0.845^b^	0.239
15	Tb927.4.5160	Tb927.8.7240	TMH/SP	0.837	0.169
17	Tb927.8.7250			0.958	0.4
20	Tb927.4.5050	Tb927.8.7380	dihydrolipoamide dehydrogenase	0.924^b^	0.303
45	Tb927.4.4540	Tb927.8.7790	LSD1 zinc finger	0.818	0.227
54	Tb927.4.4360	Tb927.8.8020	monoglyceride lipase	0.783^b^	0.199
56	Tb927.4.4330	Tb927.8.8040	diadenosine tetraphosphatase	0.781	0.214
					
**Divergent CDS/Conserved NCS**					
63	Tb927.4.4160	Tb927.8.8170	SP, CheY-like domain	0.773	0.901
65	Tb927.4.5150	Tb927.8.7240		0.901	0.983

^a^ denotes evidence for 'constant but different'   non-synonymous mutations.

^b^ denotes evidence for significant rate asymmetry between   retained paralogs.

First, for some duplicates both CDS and NCS experienced few substitutions and remained structurally conserved. Such loci included RNA polymerase IIA (**22**), dynein light chain 2B (**3**), a 3-methylcrotonyl-CoA carboxylase (**5**), as well as many other hypothetical genes where divergence in either CDS or NCS did not exceed 5%. These cases suggest that some genes involved in core cellular functions were retained to increase dose, and have been constrained by strong purifying selection. The substantial divergence of regulatory regions elsewhere makes a compelling argument for conservation of expression profile in these instances. However, high sequence identity does not preclude important structural changes, since two examples of this dynamic (**24 **and **2**) also showed non-neutral substitution patterns, with significant excesses of 'constant-but-different' amino-acid replacements. This dynamic might also occur where gene conversion homogenises gene copies in *trans*, although no evidence was seen for this here.

The second dynamic involved substantial divergence in both CDS and NCS. These cases mostly involved hypothetical genes, which is intuitive since slowly-evolving genes fundamental to cellular function are more likely to be annotated; many possessed features indicative of surface-expression, and a GTP-binding rab protein (**59**) showing 45% and 43% divergence in CDS and NCS respectively was included. Since these paralogs often diverged beyond recognition in parts of their structures, such that functional conservation is implausible, they are the best candidates for neofunctionalisation. Positive selection was detected for individual codons at some loci with this dynamic; for example **10**, **12**, **46**, **62 **and **71**. However, none of the paralogous sequences displayed a global *ω *above 1, and positive selection was not conspicuously strong. Documented cases of neofunctionalisation are sparse, perhaps because it is easier to elucidate the functional differences between gene duplicates when sequence identity remains high. One instance, the evolution of antifreeze glycoproteins from trypsinogen-like proteases in Antarctic notothenioid fish [69], demonstrates how gene duplication can be followed by fundamental remodelling of primary structures, involving the loss of functional domains and very low sequence identities, of the magnitude recorded in *T. brucei*. At least two conserved paralogs showed remodelling of this type, and so are candidates for radical new functions. First, a kinetoplastid membrane protein (**18**: Tb927.4.5120 and Tb927.8.7260) was substantially shortened on chromosome 8 relative to chromosome 4 (and orthologs in other species), involving the deletion of a large repetitive *Hint *domain and a GPI anchor signal; these features suggest a membrane-bound signalling function that has been lost by the chromosome 8 paralog. Second, a hypothetical gene (**72**: Tb927.4.3920 and Tb927.8.8340) possessed a lipid-binding domain and 12 transmembrane helices, the latter were deleted from the chromosome 8 paralog, indicating that the protein had acquired a new position within the cell.

The third dynamic combined divergence of NCS with conservation of coding regions. This kind of change might indicate where regulation of a gene has evolved without much structural change, perhaps resulting in a novel expression profile. Genes in this category are mostly metabolic enzymes, e.g., protein kinase, lipase and phosphatase; these might be expected to evolve slowly with respect to primary structure, but they offer interesting avenues for further investigation since their regulatory regions have clearly been changed considerably. Several previous cases have shown that innovation can occur through changes to regulatory domains alone. Bhushan et al. (2005) [70] described paralogous metallopeptidases in *A. thaliana *with 75.6% protein identity but differential expression in tissue-specific manner. Segregation of the ancestral function between duplicates was achieved through regulatory change (although some structural change had also altered enzyme specificity), consistent with a subfunctionalisation model. Hua et al. (2003) [71] described a similar case in humans, where *Nudt10 *and *Nudt11 *were recently duplicated phosphohydrolases, the former expressed in liver, kidney and testis and the latter restricted to brain. However, these copies were identical at the protein level. Indeed, the evolution of proteins under strong structural constraints, such as Hox genes [72,73] through changes to *cis*-acting regulatory modules provides the most comprehensive evidence that subfunctionalisation via this mechanism is a general principle.

In the final dynamic coding regions diverged, while untranslated regions remained structurally conserved. Given that non-coding regions are typically less affected by negative selection, it is intuitive that this outcome was rare. Table 5 shows that in two cases non-coding regions were conserved (90% and 98% respectively), while CDS divergence was greater, but only considerably so for one hypothetical gene (**63**), likely to be involved in signal transduction. There was no evidence of significant rate asymmetry or non-neutral substitutions between these paralogous pairs, and so it seems more likely that strong selection to preserve regulatory regions was responsible for the dynamic. Therefore, these loci might represent odd examples of structural derivation in the absence of changes to expression profiles.

In summary, the diverse divergence rates observed, (despite the time of separation being constant), the instances of contrary divergence patterns in CDS and NCS, as well as significantly asymmetric change or adaptive substitutions among many paralogous pairs, all suggest that the duplication event had equally diverse functional consequences.

## Conclusion

The *T. brucei *clade has been affected by the duplication and transposition of a large chromosomal block, perhaps due to a mistake during cell division. For events of this type, a surprisingly large proportion of gene duplicates were subsequently retained, including gene families known to be important at the host-parasite interface. Gene loss was selective, since surface-expressed genes were over-represented among conserved paralogs; this is consistent with a dosage balance hypothesis in which genes with low 'connectivity' within the cell are more likely to be preserved after segmental duplications because they are dose-insensitive. Sequence divergence of conserved paralogs followed several different evolutionary trajectories, sometimes accompanied by significant asymmetry in substitution rate and significant excesses of amino acid replacements, and generally more prone to adaptive evolution than singleton loci. Indeed, the structural change among coding and regulatory regions of conserved paralogs was often radical, providing strong indications that many of these cases involved functional change. The functional consequences of this duplication will become clear as hypothetical genes are annotated and the biological differences between paralogs investigated. However, by demonstrating considerable gene retention and structural divergence, this study has established that the duplication made a significant contribution to the genomic repertoire of *T. brucei*, relative to other trypanosomatids, and was a seminal development in its genomic evolution.

## Methods

### (a) Examination of the duplicated regions

The extent of colinearity between Chromosomes 4 and 8 in *T. brucei *was assessed using the Artemis Comparison Tool v5 (ACT [74]). A tBLASTx algorithm [75] was used to create a sequence comparison from EMBL files of the two chromosomes. The duplicated regions of Chromosomes 4 and 8 were inspected visually from the GeneDB chromosome maps page [76], to determine the gene order on each duplicon and affinity shown by each paralogous gene pair. Sequence identity was calculated as the proportion of amino acids conserved when paralogous genes were aligned in BioEdit [77]. Inspection of homologs in *Leishmania major *showed that the duplicated region in *T. brucei *corresponded to the complete Chromosome 31 in *L. major*. The gene order in *L. major *(and another related trypanosome, *T. cruzi*) was used to infer the gene order on the chromosome ancestral to the duplicons on Chromosomes 4 and 8 in *T. brucei*. If present on both duplicons and either outgroup, a locus was shared, i.e., originally single-copy and now retained as two paralogs. If present on one duplicon and either outgroup, the locus was considered to have been present on the ancestral chromosome and lost after duplication from the either duplicon. If present on one duplicon but absent from either outgroup, this was interpreted as gain of a locus after duplication. Situations were both duplicons retained a locus that was absent in both L. major and *T. cruzi *were not observed. The duplicated regions in *T. brucei *were also compared to homologous regions in its closest relative, *T. congolense*. If it could be shown that *T. congolense *possessed duplicate copies for the loci in these regions, this would show that the duplication occurred prior to the separation of these two species. Gene order along the *T. brucei *duplicons was compared with preliminary assemblies for chromosomes 4 and 8 in *T. congolense *(available from GeneDB).

### (b) Analysis of divergence of paralogous sequences

Patterns in sequence divergence post-duplication were analysed by comparing the identity shown by coding regions of paralogous gene pairs with that shown by non-coding regions. Untranslated regions of genes were identified from genomic sequence in Artemis v8 [78] after identifying the sequence motifs for spliced leader and poly-A tail additions, established previously [79]. These motifs signal the creation of individual transcripts from nascent polycistronic transcripts; they are arranged in a fairly consistent manner, with a polypyrimidine tract providing the signal for poly-A tail addition (i.e., the end of the 3' UTR), and the next downstream AG dinucleotide signalling for the addition of the spliced leader sequence (i.e., the start of the 5' UTR) [79]. Determination of these points for each paralogous gene pair allowed sequence identity to be calculated for coding, 5'UTR and 3'UTR regions respectively. If paralogous non-coding regions could be aligned, nucleotide identity was calculated from the alignment; if not, a nominal value was calculated from unaligned sequences. In both cases, sequences were trimmed to equal length since length differences would reduce sequence identity overall.

### (c) Relative rates tests

Significant departures in evolutionary rate post-duplication were identified using the relative rates test [80,81]. Paralogous gene pairs were combined in a sequence alignment with a homolog from *T. cruzi *(or where this was absent, *T. vivax*), retrieved from GeneDB, and designated as an outgroup comparison. The rates of non-synonymous substitutions per site (*D*_*n*_) between each paralog and an outgroup were compared using RRTree [82]; synonymous substitutions were not compared since these were frequently saturated over the relatively large evolutionary distances concerned. Due to the various weaknesses of the canonical, i.e., Wu-Li, relative rates test, a Bayesian approach was adopted as previously described [83]). The genetic distance between each paralog and the most recent common ancestor of all three sequences was estimated using Cadence [83]; this was calculated from 5000 Bayesian phylogenies to estimate 95% confidence intervals. Significant differences in evolutionary rate were inferred where these confidence intervals did not overlap.

### (d) Tests for non-neutral evolution

Paralogous CDS sequences were analysed for evidence of adaptive evolution using two methods. First, alignments were generated for each pair of paralogous sequences and for each singleton locus along the duplicated region, in combination with their *T. congolense *homologs. The latter were included to allow the singleton loci to be analysed and directly compared with conserved paralogs. The ratio of non-synonymous substitutions per site (*D*_*n*_) to synonymous substitutions per site (*D*_*s*_), referred to as *ω*, was calculated for each codon of each alignment using the adaptive evolution server [84], within the HYPHY platform [85]. Three methods were used to detect both positive and negative selection at individual codons: i) Single-Likelihood-Ancestor-Counting (SLAC), ii) Fixed-Effect-Likelihood (FEL) and iii) Random-Effects-Likelihood (REL), which have been described in detail elsewhere [86]. The inclusion of a *T. congolense *homolog as an outgroup sequence could introduce or obscure further evidence for selection, hence, conserved paralogs were tested again without an outgroup, using the REL method.

Second, paralogous sequences were aligned with homologs from related trypanosomatids (*T. congolense, T. vivax, T. cruzi*, as appropriate and where available) and used to create a phylogenetic tree and reconstruct ancestral sequences. This was done using CRANN [87], which applies the method of [88]. The frequencies of invariant (i.e., change once but not again) and variable (change frequently) mutations at both non-synonymous and synonymous sites were calculated along the branches leading to each paralog from their inferred ancestor. The ratios of invariant to variable mutations at non-synonymous and synonymous sites were calculated; significant differences between these values were identified using a G-test. Significant differences due to an excess of invariant mutations at non-synonymous sites ('replacement-invariable', or RI) indicate adaptive change [89,90], in essence because the ratio at synonymous sites represents the expectation under neutral conditions, and the ratio at non-synonymous sites should be not significantly different in the absence of positive selection.

### (e) Taxonomic distribution of the duplicons

Observation and analysis of the duplication event was made from the genome sequence of *T. brucei brucei *strain 927. A polymerase chain reaction (PCR) assay was applied to determine whether the duplication was also present in other strains of *T. brucei*, namely *T. b. brucei *(TSW 187), *T. b. gambiense *(Dal 972), *T. b. rhodiense *(LVH 108) and *T. evansi *(RoTat 1.2). Three locations along the duplicated region were selected where there was a shared paralog followed downstream by distinct single-copy genes (i.e., lost from one chromosome) on each duplicon. For each location, a common forward primer and dissimilar reverse primers were used to amplify the two distinct intergenic regions from each *T. brucei *subspecies. Successful amplification of products with expected size confirmed that both duplicons were present. Genomic DNA was denatured at 92°C for 2 minutes and then 35 amplification cycles were performed under the following conditions: denaturation at 92°C (30 seconds), annealing at 60°C (10 seconds) and extension at 72°C (90 seconds). The locus, forward primer, chromosome 4-specific reverse primer (with expected product size), and chromosome 8-specific reverse primer (with expected product size) are given for each location in turn. Location 1: **14 **(Tb927.4.5180 and Tb927.8.7220); 1F, TGCAACTCAGTCAGGACCCGT; 1R4 (1310 bp), TCCCAGCAACACCTTCAGTTT; 1R8 (1929 bp), TAACATTTCCACCGCTACCTG. Location 2: **25 **(Tb927.4.4990 and Tb927.8.7430); 2F, GAGCGCATCAAGGATATCCCT; 2R4 (1180 bp), GCCTCCATCAATGTTAAACCA; 2R8 (2039 bp), CTTCAAGACGAACGCAGACTC. Location 3: **55 **(Tb927.4.4330 and Tb927.8.8040); 3F, GGTCCTGAAACGGTGGTGTTT; 3R4 (1030 bp), CGTGCTGTATGGGTGATTCTT; 3R8 (1560 bp), ACAAGAAGAATGTGCCACCAC.

## Abbreviations

ACT Artemis Comparison Tool

CDS Coding sequence

FEL Fixed effects likelihood

NCS Non-coding sequence

PCR Polymerase chain reaction

REL Random effects likelihood

RI 'Replacement-invariable' mutation

SLAC Single-likelihood ancestor counting

UTR Untranscribed region

VSG Variant surface glycoprotein

WGD Whole genome duplication

## Supplementary Material

Additional data file 1 Figure S1. Comparative gene order and sequence identity between duplicons. Chromosome 4 is shown above, and running antiparallel to, chromosome 8. Scale is shown in Mbp and corresponds to positions shown on chromosome maps in GeneDB. Genes are marked on chromosomes in three colours: grey (shared paralogs on both duplicons, numbered 1 to 74), red (unilaterally lost from one duplicon) and green (unilaterally gained after duplication by one duplicon). All genes may be clicked to link to positions and gene models in GeneDB. Paralogs are linked by shaded bars that reflect amino acid sequence identity. Loci with positive identifications in GeneDB are labelled by gene name. Coloured arrows relate to significant results in sequence analyses and may be clicked to link to relevant data tables: blue (significant asymmetry in canonical relative rates test), dark blue (significant asymmetry in Bayesian relative rates test), green (significant difference in the invariable-variable mutation ratios at non-synonymous vs. synonymous sites) and red (ω > 1). Click here for file

Additional data file 2 Table S1. Paralogs retained on both duplicons: inter-chromosomal and interspecific sequence identity in CDS and NCS regions and UTR length. Click here for file

Additional data file 3 Table S2. Results of canonical relative rates tests, using the non-synonymous substitution rate per site (*D*_*n*_) since duplication, on shared, paralogous CDSs. Click here for file

Additional data file 4 Table S3. Results of Bayesian relative rates tests on shared paralogs, comparing total genetic distance to MRCA. Click here for file

Additional data file 5 Table S4. Evidence for positive and negative selection per codon for single-copy loci (i.e., duplicate lost) and retained paralogs within the duplicated region. Click here for file

Additional data file 6 Table S5. Comparisons of ratios of invariable to variable mutations at synonymous (i.e., silent, S) and non-synonymous (i.e., replacement, R) sites, between shared paralogs. Click here for file

Additional data file 7 Figure S2. PCR assay to determine the taxonomic distribution of the duplication event. To confirm the presence of both duplicons in four subspecies of *T. brucei *(*brucei *(*Tbb*), *rhodiense *(*Tbr*), *gambiense *(*Tbg*) and *evansi *(*Tev*)], three locations along the duplicated region (1–3, shown with corresponding GeneDB identifiers) were selected. In each case, a shared paralog was followed downstream by dissimilar single-copy genes on the different duplicons. Amplification of the two dissimilar intergenic regions for each location (shown at right with primer names and expected PCR products sizes) yielded the expected products from all four subspecies. Click here for file
