# Supplementary figures and images for: Evolutionary consequences of a large duplication event in Trypanosoma brucei: Chromosomes 4 and 8 are partial duplicons
Source: BMC Genomics. 2007 Nov 23;8:432. doi: 10.1186/1471-2164-8-432 (PMC2212663; doi:10.1186/1471-2164-8-432)

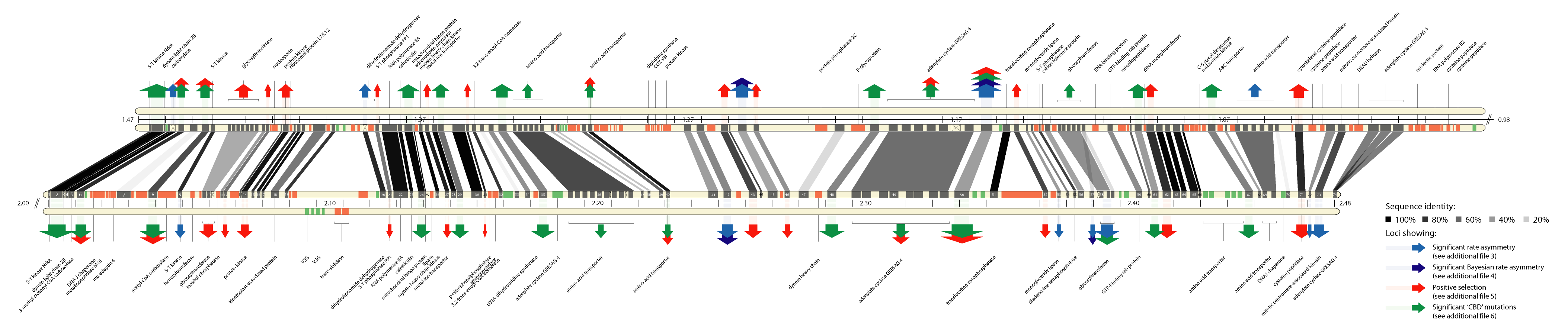

Supplement: Additional data file 1 — Figure S1. Comparative gene order and sequence identity between duplicons. Chromosome 4 is shown above, and running antiparallel to, chromosome 8. Scale is shown in Mbp and corresponds to positions shown on chromosome maps in GeneDB. Genes are marked on chromosomes in three colours: grey (shared paralogs on both duplicons, numbered 1 to 74), red (unilaterally lost from one duplicon) and green (unilaterally gained after duplication by one duplicon). All genes may be clicked to link to positions and gene models in GeneDB. Paralogs are linked by shaded bars that reflect amino acid sequence identity. Loci with positive identifications in GeneDB are labelled by gene name. Coloured arrows relate to significant results in sequence analyses and may be clicked to link to relevant data tables: blue (significant asymmetry in canonical relative rates test), dark blue (significant asymmetry in Bayesian relative rates test), green (significant difference in the invariable-variable mutation ratios at non-synonymous vs. synonymous sites) and red (ω > 1). [file 1471-2164-8-432-S1.png]

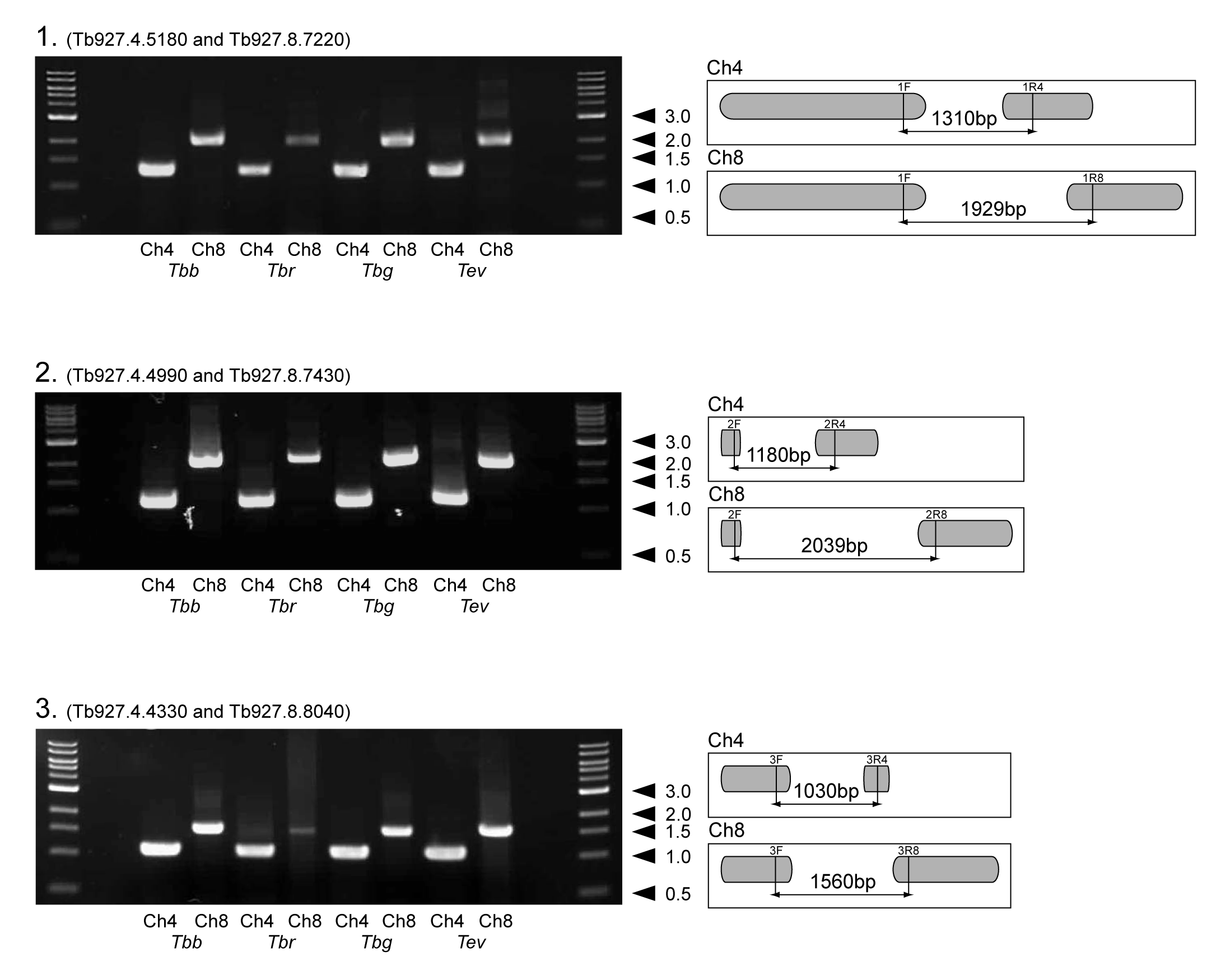

Supplement: Additional data file 7 — Figure S2. PCR assay to determine the taxonomic distribution of the duplication event. To confirm the presence of both duplicons in four subspecies of T. brucei (brucei (Tbb), rhodiense (Tbr), gambiense (Tbg) and evansi (Tev)], three locations along the duplicated region (1–3, shown with corresponding GeneDB identifiers) were selected. In each case, a shared paralog was followed downstream by dissimilar single-copy genes on the different duplicons. Amplification of the two dissimilar intergenic regions for each location (shown at right with primer names and expected PCR products sizes) yielded the expected products from all four subspecies. [file 1471-2164-8-432-S7.png]
