# Supplementary material for: Evolutionary consequences of a large duplication event in Trypanosoma brucei: Chromosomes 4 and 8 are partial duplicons
Source: BMC Genomics. 2007 Nov 23;8:432. doi: 10.1186/1471-2164-8-432 (PMC2212663; doi:10.1186/1471-2164-8-432)
Supplement: Additional data file 2 — Table S1. Paralogs retained on both duplicons: inter-chromosomal and interspecific sequence identity in CDS and NCS regions and UTR length. [file 1471-2164-8-432-S2.doc]

Table S1. Paralogs retained on both duplicons: inter-chromosomal and interspecific sequence identity in CDS and NCS regions and UTR length.

| **Locus** | **Identifer** |  | **Description** | **CDS identity** | **Tb-Tc identity** | | **5'UTR:** |  | **3'UTR** |  |
| --- | --- | --- | --- | --- | --- | --- | --- | --- | --- | --- |
|  | Chr4 | Chr8 |  |  |  |  | Identity | Length | Identity | Length |
|  |  |  |  |  |  |  |  |  |  |  |
| 1 | Tb927.4.5390 | Tb927.8.6930 | serine/threonine-protein kinase NrkA | 0.983 | 0.656 | 0.66 | 0.461 | 23 | 0.968 | 126 |
| 2 | Tb927.4.5380 | Tb927.8.6940 | alcohol dehydrogenase-like | 0.993 | 0.569 | 0.569 | 0.986 | 147 | 0.995 | 243 |
| 3 | Tb927.4.5370 | Tb927.8.6950 | dynein light chain 2B | 0.994 | 0.666 | 0.663 | 0.958 | 72 | 0.994 | 172 |
| 4 | Tb927.4.5360 | Tb927.8.6960 | TMH/SP | 0.987 | 0.645 | 0.647 | 0.949 | 118 | 0.984 | 133 |
| 5 | Tb927.4.5350 | Tb927.8.6970 | 3-methylcrotonyl-CoA carboxylase | 0.983 | 0.661 | 0.652 | 0.976 | 42 | 0.984 | 327 |
| 6 | Tb927.4.5340 | Tb927.8.6980 |  | 0.824 | 0.322 | 0.32 | 0.354 | 16 | 0.523 | 295 |
| 7 | Tb927.4.5330 | Tb927.8.7060 |  | 0.235 | - | - | 0.083 | 5 | 0.165 | 0 |
| 8 | Tb927.4.5320 | Tb927.8.7090 |  | 0.75 | 0.518 | 0.528 | 0.538 | 275 | 0.319 | 0 |
| 9 | Tb927.4.5310 | Tb927.8.7110 | serine/threonine-protein kinase A | 0.845 | 0.684 | 0.714 | 0.357 | 8 | 0.239 | ~250 |
| 10 | Tb927.4.5300 | Tb927.8.7140 | UDP-GlcNAc-dependent glycosyltransferase | 0.455 | 0.345* | 0.357* | 0.217 | 0 | 0.258 | 0 |
| 11 | Tb927.4.5230 | Tb927.8.7180 |  | 0.385 | 0.331 | 0.347 | 0.226 | 8 | 0.181 | 0 |
| 12 | Tb927.4.5220 | Tb927.8.7190 |  | 0.402 | 0.282* | 0.28* | 0.33 | 0 | 0.09 | 0 |
| 13 | Tb927.4.5190 | Tb927.8.7210 |  | 0.751 | 0.64 | 0.62 | 0.143 | 0 | 0.418 | 145 |
| 14 | Tb927.4.5180 | Tb927.8.7220 | protein kinase | 0.898 | 0.652 | 0.661 | 0.516 | 54 | 0.419 | 58 |
| 15 | Tb927.4.5160 | Tb927.8.7230 | TMH/SP | 0.837 | 0.549 | 0.546 | 0.321 | 47 | 0.169 | 0 |
| 16 | Tb927.4.5150 | Tb927.8.7240 |  | 0.901 | 0.442 | 0.419 | 0.128 | 0 | 0.983 | ~300 |
| 17 | Tb927.4.5140 | Tb927.8.7250 |  | 0.958 | 0.535 | 0.535 | 0.976 | 43 | 0.4 | 0 |
| 18 | Tb927.4.5120 | Tb927.8.7260 | kinetoplast-associated protein | 0.368 | 0.325 | 0.451 | 0.495 | 55 | 0.365 | 0 |
| 19 | Tb927.4.5100 | Tb927.8.7270 | TMH/SP | 0.827 | 0.632 | 0.637 | 0.511 | 20 | 0.41 | ~350 |
| 20 | Tb927.4.5050 | Tb927.8.7380 | dihydrolipoamide dehydrogenase | 0.924 | 0.621 | 0.647 | 0.308 | 0 | 0.303 | 18 |
| 21 | Tb927.4.5030 | Tb927.8.7390 | serine/threonine protein phosphatase PP1 | 0.959 | 0.529 | 0.521 | 0.527 | ~270 | 0.478 | 60 |
| 22 | Tb927.4.5020 | Tb927.8.7400 | RNA polymerase IIA largest subunit | 0.999 | 0.892* | 0.891* | 0.869 | 46 | 1 | 400 |
| 23 | Tb927.4.5010 | Tb927.8.7410 | calreticulin | 0.987 | 0.668 | 0.67 | 0.985 | 207 | 0.714 | ~570 |
| 24 | Tb927.4.5000 | Tb927.8.7420 | C2 calcium/lipid-binding region | 0.995 | 0.525 | 0.52 | 0.904 | 147 | 0.966 | 273 |
| 25 | Tb927.4.4990 | Tb927.8.7430 | ubiquinol-cytochrome C reductase hinge protein | 0.985 | 0.788 | 0.774 | 0.953 | 130 | 0.708 | 694 |
| 26 | Tb927.4.4970 | Tb927.8.7450 | myosin heavy chain kinase A | 0.944 | 0.608 | 0.611 | 0.925 | 67 | 0.895 | 230 |
| 27 | Tb927.4.4960 | Tb927.8.7460 | metal-ion transporter | 0.859 | 0.546 | 0.567 | 0.113 | 0 | 0.433 | 0? |
| 28 | Tb927.4.4950 | Tb927.8.7470 | aldehyde dehydrogenase, WD40 repeat | 0.988 | 0.421 | 0.419 | 0.976 | 43 | 0.701 | 310 |
| 29 | Tb927.4.4940 | Tb927.8.7480 | Phosphopantetheine attachment site | 0.491 | 0.198 | 0.195 | 0.495 | 0? | 0.427 | 0 |
| 30 | Tb927.4.4930 | Tb927.8.7490 |  | 0.987 | 0.518 | 0.52 | 0.555 | 50-260? | 0.956 | 420 |
| 31 | Tb927.4.4920 | Tb927.8.7500 | TMH/SP | 0.679 | 0.154 | 0.168 | 0.905 | 106 | 0.117 | 0 |
| 32 | Tb927.4.4910 | Tb927.8.7530 | 3,2-trans-enoyl-CoA isomerase | 0.717 | 0.62 | 0.65 | 0.532 | 80 | 0.185 | 0 |
| 33 | Tb927.4.4900 | Tb927.8.7550 |  | 0.282 | 0.181^#^ | 0.25^#^ | 0.387 | 0 | 0.16 | 0 |
| 34 | Tb927.4.4890 | Tb927.8.7560 | TMH | 0.454 | 0.311 | 0.293 | 0.103 | 0 | 0.137 | 0 |
| 35 | Tb927.4.4880 | Tb927.8.7580 | TMH/SP, Zinc finger, C3HC4 type | 0.48 | 0.263* | 0.246* | 0.077 | 0 | 0.264 | 0 |
| 36 | Tb927.4.4870 | Tb927.8.7600 | amino acid transporter | 0.702 | 0.622* | 0.628* | 0.09 | 0 | 0.182 | 0 |
| 37 | Tb927.4.4810 | Tb927.8.7710 | TMH | 0.402 | 0.194 | 0.213 | 0.155 | 0 | 0.425 | ~450? |
| 38 | Tb927.4.4790 | Tb927.8.7720 | TMH/SP | 0.391 | 0.263* | 0.267* | 0.243 | 0 | 0.288 | 0 |
| 39 | Tb927.4.4740 | Tb927.8.7730 | longevity-assurance protein | 0.73 | 0.515 | 0.517 | 0.262 | 0 | 0.207 | 0 |
| 40 | Tb927.4.4730 | Tb927.8.7740 | amino acid transporter | 0.902 | 0.719 | 0.723 | 0.876 | 73 | 0.414 | 0-450? |
| 41 | Tb927.4.4580 | Tb927.8.7750 | protein kinase | 0.446 | 0.151 | 0.155 | 0.21 | 0 | 0.422 | 0? |
| 42 | Tb927.4.4570 | Tb927.8.7760 |  | 0.516 | 0.453 | 0.366 | 0.247 | 0 | 0.436 | 0 |
| 43 | Tb927.4.4550 | Tb927.8.7780 | GPI anchor | 0.74 | 0.585 | 0.567 | 0.251 | 0 | 0.269 | 0 |
| 44 | Tb927.4.4540 | Tb927.8.7790 | LSD1 zinc finger | 0.818 | 0.609 | 0.632 | 0.32 | 0? | 0.227 | 0 |
| 45 | Tb927.4.4530 | Tb927.8.7800 |  | 0.407 | 0.218* | 0.242* | 0.396 | 80 | 0.47 | 500? |
| 46 | Tb927.4.4520 | Tb927.8.7820 | cold-shock protein, DNA-binding | 0.434 | 0.262 | 0.229 | 0.48 | 410 | 0.222 | 15 |
| 47 | Tb927.4.4500 | Tb927.8.7830 |  | 0.34 | 0.154* | 0.163* | 0.211 | 0 | 0.25 | 0 |
| 48 | Tb927.4.4480 | Tb927.8.7850 |  | 0.526 | 0.257 | 0.258 | 0.263 | 0 | 0.25 | 0 |
| 49 | Tb927.4.4470 | Tb927.8.7860 | adenylate cyclase GRESAG 4 | 0.592 | 0.467* | 0.467* | - | - | 0.131 | 0 |
| 50 | Tb927.4.4400 | Tb927.8.7950 |  | 0.433 | 0.239 | 0.261 | 0.156 | 0 | 0.25 | 0 |
| 51 | Tb927.4.4380 | Tb927.8.7980 | V-type H(+)-translocating pyrophosphatase | 0.992 | 0.739 | 0.739 | 0.621 | 275 | 0.484 | ~100 |
| 52 | Tb927.4.4370 | Tb927.8.8000 |  | 0.701 | 0.532 | 0.501 | 0.565 | 40 | 0.278 | 0 |
| 53 | Tb927.4.4360 | Tb927.8.8020 | monoglyceride lipase | 0.783 | 0.616 | 0.553 | 0.098 | 0 | 0.199 | 0 |
| 54 | Tb927.4.4350 | Tb927.8.8030 | TMH/SP | 0.541 | 0.432 | 0.42 | 0.256 | 0 | 0.15 | 0 |
| 55 | Tb927.4.4330 | Tb927.8.8040 | diadenosine tetraphosphatase | 0.781 | 0.581 | 0.607 | 0.27 | 0 | 0.214 | 0 |
| 56 | Tb927.4.4310 | Tb927.8.8050 | spectrin repeat | 0.366 | 0.197 | 0.274 | 0.204 | 0 | 0.152 | 0 |
| 57 | Tb927.4.4290 | Tb927.8.8090 | UDP-GlcNAc-dependent glycosyltransferase | 0.519 | 0.438 | 0.416 | 0.192 | 0 | 0.222 | 0 |
| 58 | Tb927.4.4240 | Tb927.8.8070 |  |  |  |  |  |  |  |  |
| 59 | Tb927.4.4220 | Tb927.8.8140 | small GTP-binding rab protein | 0.452 | 0.267 | 0.242 | 0.529 | 17 | 0.433 | ~500? |
| 60 | Tb927.4.4190 | Tb927.8.8150 | C2 calcium/lipid-binding region | 0.59 | 0.347 | 0.416 | 0.484 | 66 | 0.241 | 0 |
| 61 | Tb927.4.4180 | Tb927.8.8160 |  | 0.48 | 0.228 | 0.22 | 0.196 | 0 | 0.186 | 0 |
| 62 | Tb927.4.4160 | Tb927.8.8170 | CheY-like domain | 0.773 | 0.448 | 0.447 | 0.465 | 43 | 0.901 | 400 |
| 63 | Tb927.4.4150 | Tb927.8.8180 |  | 0.991 | 0.56 | 0.56 | 0.955 | 249 | 0.995 | 240 |
| 64 | Tb927.4.4140 | Tb927.8.8190 |  | 0.986 | 0.503 | 0.496 | 0.987 | 324 | 0.981 | 642 |
| 65 | Tb927.4.4130 | Tb927.8.8200 | prefoldin domain | 0.948 | 0.574 | 0.581 | 0.783 | 160 | 0.658 | 600 |
| 66 | Tb927.4.4120 | Tb927.8.8210 |  | 0.984 | 0.757 | 0.757 | 0.95 | 223 | 0.982 | 175 |
| 67 | Tb927.4.4060 | Tb927.8.8270 | 3'5'-cyclic nucleotide phosphodiesterase | 0.533 | 0.265 | 0.268 | 0.391 | 0 | 0.15 | 0 |
| 68 | Tb927.4.4040 | Tb927.8.8280 |  | 0.436 | 0.262 | 0.278 | 0.453 | 160? | 0.209 | 0 |
| 69 | Tb927.4.4020 | Tb927.8.8290 | amino acid transporter AATP5 | 0.734 | - | - | 0.261 | 0 | 0.241 | 0 |
| 70 | Tb927.4.3970 | Tb927.8.8320 |  | 0.277 | 0.203 | 0.158 | 0.27 | 0 | 0.302 | 0 |
| 71 | Tb927.4.3950 | Tb927.8.8330 | cytoskeleton-associated protein CAP5.5 | 0.748 | 0.414 | 0.408 | 0.298 | 0 | 0.258 | 0 |
| 72 | Tb927.4.3920 | Tb927.8.8340 | TMH, CRAL-TRIO lipid binding domain | 0.727 | 0.609 | 0.559 | 0.166 | 0 | 0.276 | 0 |
| 73 | Tb927.4.3910 | Tb927.8.8350 | mitotic centromer-associated kinesin | 0.686 | 0.43 | 0.432 | 0.473 | 190 | - | - |
| 74 | Tb927.4.3880 | Tb927.8.8360 | receptor-type adenylate cyclase GRESAG 4 | 0.487 | 0.133* | 0.145* | 0.312 | 0 | - | - |

Alternative interspecific comparisons are denoted by an asterisk (*T. vivax* in place of *T. cruzi*) or a crosshatch # (*T. congolense*).
