# Supplementary material for: Evolutionary consequences of a large duplication event in Trypanosoma brucei: Chromosomes 4 and 8 are partial duplicons
Source: BMC Genomics. 2007 Nov 23;8:432. doi: 10.1186/1471-2164-8-432 (PMC2212663; doi:10.1186/1471-2164-8-432)
Supplement: Additional data file 3 — Table S2. Results of canonical relative rates tests, using the non-synonymous substitution rate per site (Dn) since duplication, on shared, paralogous CDSs. [file 1471-2164-8-432-S3.doc]

Table S2. Results of canonical relative rates tests, using the non-synonymous substitution rate per site (*D_n_*) since duplication, on shared, paralogous CDSs.

| **Locus** | **Identifier** |  | **Description** | **RRT:** |  |  |  |  |  |  |
| --- | --- | --- | --- | --- | --- | --- | --- | --- | --- | --- |
|  | Chr4 | Chr8 |  | # Sites | *D_n_*1 | *D_n_*2 | d*D_n_* | SD | Ratio | P |
|  |  |  |  |  |  |  |  |  |  |  |
| 1 | Tb927.4.5390 | Tb927.8.6930 | serine/threonine-protein kinase NrkA | 861.759 | 0.23815 | 0.23429 | 0.00386 | 0.00462 | 0.83696 | 0.402624 |
| 2 | Tb927.4.5380 | Tb927.8.6940 | alcohol dehydrogenase-like | 2806.68 | 0.39452 | 0.39655 | -0.002 | 0.00151 | -1.3448 | 0.178691 |
| 3 | Tb927.4.5370 | Tb927.8.6950 | dynein light chain 2B | 226.955 | 0.22818 | 0.23459 | -0.0064 | 0.00593 | -1.0806 | 0.279901 |
| 4 | Tb927.4.5360 | Tb927.8.6960 | TMH/SP | 752.467 | 0.25589 | 0.24967 | 0.00622 | 0.00472 | 1.31813 | 0.18747 |
| 5 | Tb927.4.5350 | Tb927.8.6970 | 3-methylcrotonyl-CoA carboxylase | **1361.46** | **0.24051** | **0.25273** | **-0.0122** | **0.00437** | **-2.7971** | **0.005162** |
| 6 | Tb927.4.5340 | Tb927.8.6980 |  | 1409.47 | 0.83052 | 0.8664 | -0.0359 | 0.0223 | -1.6088 | 0.107677 |
| 7 | Tb927.4.5330 | Tb927.8.7060 |  | - | - | - | - | - | - | - |
| 8 | Tb927.4.5320 | Tb927.8.7090 |  | 1664.33 | 0.5199 | 0.4944 | 0.0255 | 0.0172 | 1.48233 | 0.138261 |
| 9 | Tb927.4.5310 | Tb927.8.7110 | serine/threonine-protein kinase A | **887.089** | **0.18419** | **0.14118** | **0.04301** | **0.01114** | **3.86206** | **0.000114** |
| 10 | Tb927.4.5300 | Tb927.8.7140 | UDP-GlcNAc-dependent glycosyltransferase | 599.008 | 0.55451 | 0.52875 | 0.02576 | 0.03902 | 0.66013 | 0.509176 |
| 11 | Tb927.4.5230 | Tb927.8.7180 |  | 377.363 | 0.85512 | 0.82128 | 0.03384 | 0.0949 | 0.35662 | 0.721379 |
| 12 | Tb927.4.5220 | Tb927.8.7190 |  | 637.399 | 0.75909 | 0.76171 | -0.0026 | 0.05477 | -0.0478 | 0.961911 |
| 13 | Tb927.4.5190 | Tb927.8.7210 |  | 308.227 | 0.23902 | 0.26751 | -0.0285 | 0.02534 | -1.1246 | 0.26076 |
| 14 | Tb927.4.5180 | Tb927.8.7220 | protein kinase | 1261.67 | 0.24568 | 0.23619 | 0.00949 | 0.00798 | 1.1899 | 0.234 |
| 15 | Tb927.4.5160 | Tb927.8.7230 | TMH/SP | 534.671 | 0.42149 | 0.42665 | -0.0052 | 0.01996 | -0.2587 | 0.795873 |
| 16 | Tb927.4.5150 | Tb927.8.7240 |  | 709.401 | 0.5505 | 0.57012 | -0.0196 | 0.01671 | -1.1748 | 0.240099 |
| 17 | Tb927.4.5140 | Tb927.8.7250 |  | 496.782 | 0.43039 | 0.43472 | -0.0043 | 0.00957 | -0.452 | 0.651301 |
| 18 | Tb927.4.5120 | Tb927.8.7260 | kinetoplast-associated protein | 1875.9 | 0.53165 | 0.51465 | 0.01699 | 0.02281 | 0.74518 | 0.456168 |
| 19 | Tb927.4.5100 | Tb927.8.7270 | TMH/SP | 809.013 | 0.28109 | 0.27137 | 0.00972 | 0.01422 | 0.68367 | 0.494189 |
| 20 | Tb927.4.5050 | Tb927.8.7380 | dihydrolipoamide dehydrogenase | **1084.98** | **0.25477** | **0.2679** | **-0.0131** | **0.00496** | **-2.6473** | **0.00812** |
| 21 | Tb927.4.5030 | Tb927.8.7390 | serine/threonine protein phosphatase PP1 | 693.823 | 0.19649 | 0.19538 | 0.00112 | 0.00759 | 0.14735 | 0.882857 |
| 22 | Tb927.4.5020 | Tb927.8.7400 | RNA polymerase IIA largest subunit | 2156.57 | 0.07077 | 0.07127 | -0.0005 | 0.00038 | -1.2961 | 0.194955 |
| 23 | Tb927.4.5010 | Tb927.8.7410 | calreticulin | 822.231 | 0.2176 | 0.21589 | 0.00171 | 0.00356 | 0.48198 | 0.62982 |
| 24 | Tb927.4.5000 | Tb927.8.7420 | C2 calcium/lipid-binding region | 1624.06 | 0.34517 | 0.34616 | -0.001 | 0.00184 | -0.5366 | 0.591568 |
| 25 | Tb927.4.4990 | Tb927.8.7430 | ubiquinol-cytochrome C reductase hinge protein | 149.799 | 0.1215 | 0.12978 | -0.0083 | 0.00807 | -1.0247 | 0.305493 |
| 26 | Tb927.4.4970 | Tb927.8.7450 | myosin heavy chain kinase A | 650.134 | 0.31043 | 0.30621 | 0.00422 | 0.00897 | 0.47056 | 0.637955 |
| 27 | Tb927.4.4960 | Tb927.8.7460 | metal-ion transporter | 889.937 | 0.30963 | 0.28856 | 0.02107 | 0.01263 | 1.66834 | 0.095255 |
| 28 | Tb927.4.4950 | Tb927.8.7470 | aldehyde dehydrogenase, WD40 repeat | 805.493 | 0.39951 | 0.40469 | -0.0052 | 0.00319 | -1.6244 | 0.104309 |
| 29 | Tb927.4.4940 | Tb927.8.7480 | Phosphopantetheine attachment site | 885.503 | 0.80794 | 0.86203 | -0.0541 | 0.04019 | -1.3458 | 0.178 |
| 30 | Tb927.4.4930 | Tb927.8.7490 |  | 2727.57 | 0.34877 | 0.35092 | -0.0022 | 0.00201 | -1.0729 | 0.283348 |
| 31 | Tb927.4.4920 | Tb927.8.7500 | TMH/SP | 396.251 | 1.06249 | 1.1807 | -0.1182 | 0.07098 | -1.6654 | 0.095841 |
| 32 | Tb927.4.4910 | Tb927.8.7530 | 3,2-trans-enoyl-CoA isomerase | 704.193 | 0.21141 | 0.23766 | -0.0263 | 0.01575 | -1.6665 | 0.095623 |
| 33 | Tb927.4.4900 | Tb927.8.7550 |  | - | - | - | - | - | - | - |
| 34 | Tb927.4.4890 | Tb927.8.7560 | TMH | 795.2 | 0.49294 | 0.53918 | -0.0462 | 0.03022 | -1.5304 | 0.125928 |
| 35 | Tb927.4.4880 | Tb927.8.7580 | TMH/SP, Zinc finger, C3HC4 type | 1352.33 | 0.7024 | 0.68672 | 0.01568 | 0.02949 | 0.53171 | 0.594929 |
| 36 | Tb927.4.4870 | Tb927.8.7600 | amino acid transporter | 920.322 | 0.30014 | 0.28793 | 0.01221 | 0.01857 | 0.65727 | 0.511014 |
| 37 | Tb927.4.4810 | Tb927.8.7710 | TMH | 429.701 | 0.95031 | 0.90033 | 0.04999 | 0.08973 | 0.55707 | 0.577484 |
| 38 | Tb927.4.4790 | Tb927.8.7720 | TMH/SP | 438.941 | 0.83773 | 0.88412 | -0.0464 | 0.07729 | -0.6003 | 0.548313 |
| 39 | Tb927.4.4740 | Tb927.8.7730 | longevity-assurance protein | 765.71 | 0.34374 | 0.34783 | -0.0041 | 0.02117 | -0.1934 | 0.8466872 |
| 40 | Tb927.4.4730 | Tb927.8.7740 | amino acid transporter | 931.352 | 0.18435 | 0.18608 | -0.0017 | 0.00851 | -0.2035 | 0.83876210 |
| 41 | Tb927.4.4580 | Tb927.8.7750 | protein kinase | 1472.3 | 1.02033 | 1.0354 | -0.0151 | 0.04616 | -0.3265 | 0.744053 |
| 42 | Tb927.4.4570 | Tb927.8.7760 |  | **1723.4** | **0.43862** | **0.60616** | **-0.1675** | **0.02294** | **-7.3021** | **1.00E-07411** |
| 43 | Tb927.4.4550 | Tb927.8.7780 | GPI anchor | 1551.84 | 0.26259 | 0.2827 | -0.0201 | 0.01231 | -1.6333 | 0.1024165 |
| 44 | Tb927.4.4540 | Tb927.8.7790 | LSD1 zinc finger | 240.537 | 0.24797 | 0.23886 | 0.00911 | 0.02571 | 0.35447 | 0.7229886 |
| 45 | Tb927.4.4530 | Tb927.8.7800 |  | **1881.03** | **0.88552** | **0.76386** | **0.12166** | **0.03476** | **3.49964** | **0.000477** |
| 46 | Tb927.4.4520 | Tb927.8.7820 | cold-shock protein, DNA-binding | 861.098 | 0.7824 | 0.84927 | -0.0669 | 0.04491 | -1.489 | 0.1364888 |
| 47 | Tb927.4.4500 | Tb927.8.7830 |  | 2127.12 | 1.40996 | 1.3923 | 0.01765 | 0.07312 | 0.24139 | 0.8092549 |
| 48 | Tb927.4.4480 | Tb927.8.7850 |  | 1420.07 | 0.79081 | 0.73828 | 0.05252 | 0.03053 | 1.7205 |  |
| 49 | Tb927.4.4470 | Tb927.8.7860 | adenylate cyclase GRESAG 4 | 1440.72 | 0.46322 | 0.46768 | -0.0045 | 0.02078 | -0.2148 | 0.829894 |
| 50 | Tb927.4.4400 | Tb927.8.7950 |  | **3307.99** | **0.93875** | **0.86283** | **0.07592** | **0.02782** | **2.72919** | **0.006355** |
| 51 | Tb927.4.4380 | Tb927.8.7980 | V-type H(+)-translocating pyrophosphatase | 1648.62 | 0.17132 | 0.16909 | 0.00223 | 0.00166 | 1.34615 | 0.178262 |
| 52 | Tb927.4.4370 | Tb927.8.8000 |  | 1047.39 | 0.34496 | 0.37687 | -0.0319 | 0.01758 | -1.8156 | 0.069441 |
| 53 | Tb927.4.4360 | Tb927.8.8020 | monoglyceride lipase | **610.949** | **0.3002** | **0.343** | **-0.0428** | **0.01825** | **-2.3457** | **0.019002** |
| 54 | Tb927.4.4350 | Tb927.8.8030 | TMH/SP | 469.833 | 0.54481 | 0.58689 | -0.0421 | 0.04789 | -0.8788 | 0.379516 |
| 55 | Tb927.4.4330 | Tb927.8.8040 | diadenosine tetraphosphatase | 477.063 | 0.31124 | 0.29055 | 0.02069 | 0.02205 | 0.93818 | 0.34816 |
| 56 | Tb927.4.4310 | Tb927.8.8050 | spectrin repeat | 1169.83 | 0.90717 | 0.82184 | 0.08533 | 0.04468 | 1.90967 | 0.056184 |
| 57 | Tb927.4.4290 | Tb927.8.8090 | UDP-GlcNAc-dependent glycosyltransferase | **753.29** | **0.49738** | **0.56549** | **-0.0681** | **0.03449** | **-1.9745** | **0.048332** |
| 58 | Tb927.4.4240 | Tb927.8.8070 |  | **151.469** | **0.54682** | **0.93046** | **-0.3836** | **0.11732** | **-3.2701** | **0.001079** |
| 59 | Tb927.4.4220 | Tb927.8.8140 | small GTP-binding rab protein | 943.833 | 0.72212 | 0.74895 | -0.0268 | 0.03921 | -0.6843 | 0.493821 |
| 60 | Tb927.4.4190 | Tb927.8.8150 | C2 calcium/lipid-binding region | 311.7 | 0.34125 | 0.28439 | 0.05686 | 0.03179 | 1.78853 | 0.073699 |
| 61 | Tb927.4.4180 | Tb927.8.8160 |  | 1512.9 | 0.90029 | 0.94269 | -0.0424 | 0.03924 | -1.0803 | 0.280003 |
| 62 | Tb927.4.4160 | Tb927.8.8170 | CheY-like domain | 1623.6 | 0.45398 | 0.45512 | -0.0011 | 0.01357 | -0.0841 | 0.933004 |
| 63 | Tb927.4.4150 | Tb927.8.8180 |  | 1744.58 | 0.33005 | 0.33202 | -0.002 | 0.00214 | -0.9236 | 0.355724 |
| 64 | Tb927.4.4140 | Tb927.8.8190 |  | 490.189 | 0.39873 | 0.4058 | -0.0071 | 0.00675 | -1.0466 | 0.295299 |
| 65 | Tb927.4.4130 | Tb927.8.8200 | prefoldin domain | 1767.96 | 0.33451 | 0.32191 | 0.0126 | 0.00732 | 1.72226 | 0.085032 |
| 66 | Tb927.4.4120 | Tb927.8.8210 |  | 261.906 | 0.14769 | 0.14478 | 0.00291 | 0.00653 | 0.44598 | 0.655617 |
| 67 | Tb927.4.4060 | Tb927.8.8270 | 3'5'-cyclic nucleotide phosphodiesterase | 1800.31 | 0.82047 | 0.85388 | -0.0334 | 0.03314 | -1.0083 | 0.313343 |
| 68 | Tb927.4.4040 | Tb927.8.8280 |  | 491.108 | 0.75961 | 0.71517 | 0.04443 | 0.05667 | 0.7841 | 0.432986 |
| 69 | Tb927.4.4020 | Tb927.8.8290 | amino acid transporter AATP5 | **944.058** | **0.2199** | **0.18246** | **0.03744** | **0.01494** | **2.50575** | **0.012225** |
| 70 | Tb927.4.3970 | Tb927.8.8320 |  | 819.086 | 0.949 | 1.03515 | -0.0862 | 0.07228 | -1.192 | 0.233286 |
| 71 | Tb927.4.3950 | Tb927.8.8330 | cytoskeleton-associated protein CAP5.5 | 1615.93 | 0.4966 | 0.50002 | -0.0034 | 0.01332 | -0.2567 | 0.797425 |
| 72 | Tb927.4.3920 | Tb927.8.8340 | TMH, CRAL-TRIO lipid binding domain | **564.376** | **0.26532** | **0.36032** | **-0.095** | **0.02302** | **-4.1268** | **3.87E-05** |
| 73 | Tb927.4.3910 | Tb927.8.8350 | mitotic centromer-associated kinesin | **1339.89** | **0.49702** | **0.53592** | **-0.0389** | **0.01817** | **-2.1408** | **0.032297** |
| 74 | Tb927.4.3880 | Tb927.8.8360 | receptor-type adenylate cyclase GRESAG 4 | 864.712 | 1.29452 | 1.23513 | 0.05939 | 0.09926 | 0.59838 | 0.549591 |

Significant results are shown in bold.

Missing values are denoted by a dash – and indicate the absence of an appropriate outgroup.
