# Supplementary material for: Evolutionary consequences of a large duplication event in Trypanosoma brucei: Chromosomes 4 and 8 are partial duplicons
Source: BMC Genomics. 2007 Nov 23;8:432. doi: 10.1186/1471-2164-8-432 (PMC2212663; doi:10.1186/1471-2164-8-432)
Supplement: Additional data file 4 — Table S3. Results of Bayesian relative rates tests on shared paralogs, comparing total genetic distance to MRCA. [file 1471-2164-8-432-S4.doc]

Table S3. Results of Bayesian relative rates tests on shared paralogs, comparing total genetic distance to MRCA.

| **Locus** | **Identifier** |  | **Description** | **Sequence 1:** | |  |  | **Sequence 2:** | |  |  |
| --- | --- | --- | --- | --- | --- | --- | --- | --- | --- | --- | --- |
|  | Chr4 | Chr8 |  | Average | SD | Range |  | Average | SD | Range |  |
|  |  |  |  |  |  |  |  |  |  |  |  |
| 1 | Tb927.4.5390 | Tb927.8.6930 | serine/threonine-protein kinase NrkA | 0.258 | 0.019 | 0.220 | 0.297 | 0.250 | 0.019 | 0.212 | 0.288 |
| 2 | Tb927.4.5380 | Tb927.8.6940 | alcohol dehydrogenase-like | 0.290 | 0.014 | 0.262 | 0.319 | 0.289 | 0.014 | 0.261 | 0.318 |
| 3 | Tb927.4.5370 | Tb927.8.6950 | dynein light chain 2B | 0.212 | 0.035 | 0.143 | 0.282 | 0.212 | 0.035 | 0.143 | 0.281 |
| 4 | Tb927.4.5360 | Tb927.8.6960 | TMH/SP | 0.245 | 0.021 | 0.203 | 0.287 | 0.243 | 0.021 | 0.201 | 0.285 |
| 5 | Tb927.4.5350 | Tb927.8.6970 | 3-methylcrotonyl-CoA carboxylase | 0.223 | 0.015 | 0.194 | 0.253 | 0.238 | 0.015 | 0.208 | 0.268 |
| 6 | Tb927.4.5340 | Tb927.8.6980 |  | 0.525 | 0.040 | 0.444 | 0.606 | 0.495 | 0.039 | 0.417 | 0.573 |
| 7 | Tb927.4.5330 | Tb927.8.7060 |  | - | - | - | - | - | - | - | - |
| 8 | Tb927.4.5320 | Tb927.8.7090 |  | 0.458 | 0.023 | 0.412 | 0.504 | 0.438 | 0.022 | 0.395 | 0.482 |
| 9 | Tb927.4.5310 | Tb927.8.7110 | serine/threonine-protein kinase A | 0.266 | 0.019 | 0.229 | 0.303 | 0.215 | 0.016 | 0.182 | 0.248 |
| 10 | Tb927.4.5300 | Tb927.8.7140 | UDP-GlcNAc-dependent glycosyltransferase | 0.328 | 0.033 | 0.263 | 0.394 | 0.245 | 0.029 | 0.188 | 0.303 |
| 11 | Tb927.4.5230 | Tb927.8.7180 |  | 0.441 | 0.060 | 0.322 | 0.561 | 0.452 | 0.063 | 0.326 | 0.578 |
| 12 | Tb927.4.5220 | Tb927.8.7190 |  | 0.435 | 0.047 | 0.341 | 0.529 | 0.492 | 0.051 | 0.390 | 0.595 |
| 13 | Tb927.4.5190 | Tb927.8.7210 |  | 0.223 | 0.034 | 0.155 | 0.291 | 0.243 | 0.035 | 0.173 | 0.314 |
| 14 | Tb927.4.5180 | Tb927.8.7220 | protein kinase | 0.221 | 0.016 | 0.189 | 0.252 | 0.223 | 0.016 | 0.192 | 0.255 |
| 15 | Tb927.4.5160 | Tb927.8.7230 | TMH/SP | 0.310 | 0.030 | 0.249 | 0.370 | 0.343 | 0.032 | 0.279 | 0.407 |
| 16 | Tb927.4.5150 | Tb927.8.7240 |  | 0.443 | 0.041 | 0.360 | 0.526 | 0.470 | 0.043 | 0.385 | 0.555 |
| 17 | Tb927.4.5140 | Tb927.8.7250 |  | 0.264 | 0.028 | 0.208 | 0.320 | 0.238 | 0.027 | 0.185 | 0.292 |
| 18 | Tb927.4.5120 | Tb927.8.7260 | kinetoplast-associated protein | 0.513 | 0.026 | 0.461 | 0.566 | 0.406 | 0.021 | 0.364 | 0.449 |
| 19 | Tb927.4.5100 | Tb927.8.7270 | TMH/SP | 0.282 | 0.022 | 0.239 | 0.325 | 0.257 | 0.021 | 0.215 | 0.298 |
| 20 | Tb927.4.5050 | Tb927.8.7380 | dihydrolipoamide dehydrogenase | 0.243 | 0.017 | 0.210 | 0.276 | 0.253 | 0.017 | 0.219 | 0.287 |
| 21 | Tb927.4.5030 | Tb927.8.7390 | serine/threonine protein phosphatase PP1 | 0.196 | 0.018 | 0.159 | 0.232 | 0.208 | 0.019 | 0.170 | 0.245 |
| 22 | Tb927.4.5020 | Tb927.8.7400 | RNA polymerase IIA largest subunit | 0.129 | 0.008 | 0.113 | 0.144 | 0.130 | 0.008 | 0.115 | 0.146 |
| 23 | Tb927.4.5010 | Tb927.8.7410 | calreticulin | 0.178 | 0.018 | 0.142 | 0.214 | 0.178 | 0.018 | 0.142 | 0.213 |
| 24 | Tb927.4.5000 | Tb927.8.7420 | C2 calcium/lipid-binding region | 0.263 | 0.017 | 0.229 | 0.297 | 0.264 | 0.017 | 0.230 | 0.298 |
| 25 | Tb927.4.4990 | Tb927.8.7430 | ubiquinol-cytochrome C reductase hinge protein | 0.181 | 0.036 | 0.109 | 0.253 | 0.186 | 0.036 | 0.114 | 0.259 |
| 26 | Tb927.4.4970 | Tb927.8.7450 | myosin heavy chain kinase A | 0.275 | 0.024 | 0.228 | 0.323 | 0.266 | 0.023 | 0.220 | 0.312 |
| 27 | Tb927.4.4960 | Tb927.8.7460 | metal-ion transporter | 0.336 | 0.025 | 0.286 | 0.386 | 0.297 | 0.023 | 0.250 | 0.344 |
| 28 | Tb927.4.4950 | Tb927.8.7470 | aldehyde dehydrogenase, WD40 repeat | 0.367 | 0.029 | 0.309 | 0.425 | 0.370 | 0.029 | 0.312 | 0.428 |
| 29 | Tb927.4.4940 | Tb927.8.7480 | Phosphopantetheine attachment site | 0.378 | 0.042 | 0.294 | 0.462 | 0.441 | 0.045 | 0.351 | 0.531 |
| 30 | Tb927.4.4930 | Tb927.8.7490 |  | 0.226 | 0.011 | 0.203 | 0.248 | 0.225 | 0.011 | 0.202 | 0.248 |
| 31 | Tb927.4.4920 | Tb927.8.7500 | TMH/SP | 0.456 | 0.067 | 0.323 | 0.589 | 0.493 | 0.068 | 0.356 | 0.629 |
| 32 | Tb927.4.4910 | Tb927.8.7530 | 3,2-trans-enoyl-CoA isomerase | 0.280 | 0.023 | 0.235 | 0.325 | 0.291 | 0.023 | 0.244 | 0.337 |
| 33 | Tb927.4.4900 | Tb927.8.7550 |  | 0.710 | 0.074 | 0.561 | 0.859 | 0.563 | 0.064 | 0.434 | 0.692 |
| 34 | Tb927.4.4890 | Tb927.8.7560 | TMH | 0.486 | 0.034 | 0.417 | 0.555 | 0.494 | 0.036 | 0.423 | 0.566 |
| 35 | Tb927.4.4880 | Tb927.8.7580 | TMH/SP, Zinc finger, C3HC4 type | 0.540 | 0.034 | 0.473 | 0.608 | 0.521 | 0.033 | 0.455 | 0.588 |
| 36 | Tb927.4.4870 | Tb927.8.7600 | amino acid transporter | 0.539 | 0.027 | 0.485 | 0.594 | 0.493 | 0.025 | 0.443 | 0.543 |
| 37 | Tb927.4.4810 | Tb927.8.7710 | TMH | 0.318 | 0.023 | 0.271 | 0.365 | 0.308 | 0.022 | 0.263 | 0.353 |
| 38 | Tb927.4.4790 | Tb927.8.7720 | TMH/SP | 0.452 | 0.064 | 0.325 | 0.580 | 0.494 | 0.071 | 0.351 | 0.636 |
| 39 | Tb927.4.4740 | Tb927.8.7730 | longevity-assurance protein | 0.560 | 0.066 | 0.428 | 0.693 | 0.426 | 0.046 | 0.333 | 0.518 |
| 40 | Tb927.4.4730 | Tb927.8.7740 | amino acid transporter | 0.349 | 0.025 | 0.298 | 0.400 | 0.363 | 0.026 | 0.311 | 0.415 |
| 41 | Tb927.4.4580 | Tb927.8.7750 | protein kinase | 0.433 | 0.033 | 0.366 | 0.500 | 0.440 | 0.034 | 0.372 | 0.507 |
| 42 | Tb927.4.4570 | Tb927.8.7760 |  | 0.448 | 0.022 | 0.404 | 0.492 | 0.577 | 0.027 | **0.524** | **0.630** |
| 43 | Tb927.4.4550 | Tb927.8.7780 | GPI anchor | 0.233 | 0.015 | 0.202 | 0.264 | 0.247 | 0.016 | 0.214 | 0.279 |
| 44 | Tb927.4.4540 | Tb927.8.7790 | LSD1 zinc finger | 0.370 | 0.046 | 0.278 | 0.463 | 0.334 | 0.043 | 0.247 | 0.421 |
| 45 | Tb927.4.4530 | Tb927.8.7800 |  | 0.491 | 0.028 | **0.435** | **0.548** | 0.334 | 0.024 | 0.286 | 0.382 |
| 46 | Tb927.4.4520 | Tb927.8.7820 | cold-shock protein, DNA-binding | 0.544 | 0.054 | 0.436 | 0.651 | 0.536 | 0.053 | 0.430 | 0.643 |
| 47 | Tb927.4.4500 | Tb927.8.7830 |  | 0.540 | 0.047 | 0.446 | 0.634 | 0.609 | 0.051 | 0.507 | 0.710 |
| 48 | Tb927.4.4480 | Tb927.8.7850 |  | 0.497 | 0.033 | 0.432 | 0.562 | 0.501 | 0.034 | 0.434 | 0.568 |
| 49 | Tb927.4.4470 | Tb927.8.7860 | adenylate cyclase GRESAG 4 | 0.407 | 0.022 | 0.363 | 0.451 | 0.382 | 0.021 | 0.340 | 0.424 |
| 50 | Tb927.4.4400 | Tb927.8.7950 |  | 0.642 | 0.027 | **0.588** | **0.696** | 0.510 | 0.023 | 0.464 | 0.556 |
| 51 | Tb927.4.4380 | Tb927.8.7980 | V-type H(+)-translocating pyrophosphatase | 0.173 | 0.012 | 0.149 | 0.196 | 0.172 | 0.012 | 0.149 | 0.195 |
| 52 | Tb927.4.4370 | Tb927.8.8000 |  | 0.300 | 0.022 | 0.257 | 0.344 | 0.353 | 0.023 | 0.307 | 0.400 |
| 53 | Tb927.4.4360 | Tb927.8.8020 | monoglyceride lipase | 0.280 | 0.026 | 0.228 | 0.332 | 0.355 | 0.030 | 0.296 | 0.415 |
| 54 | Tb927.4.4350 | Tb927.8.8030 | TMH/SP | 0.381 | 0.040 | 0.300 | 0.462 | 0.404 | 0.042 | 0.320 | 0.489 |
| 55 | Tb927.4.4330 | Tb927.8.8040 | diadenosine tetraphosphatase | 0.259 | 0.027 | 0.204 | 0.313 | 0.276 | 0.029 | 0.219 | 0.334 |
| 56 | Tb927.4.4310 | Tb927.8.8050 | spectrin repeat | 0.519 | 0.041 | 0.437 | 0.600 | 0.482 | 0.040 | 0.402 | 0.562 |
| 57 | Tb927.4.4290 | Tb927.8.8090 | UDP-GlcNAc-dependent glycosyltransferase | 0.424 | 0.036 | 0.352 | 0.496 | 0.451 | 0.038 | 0.374 | 0.527 |
| 58 | Tb927.4.4240 | Tb927.8.8070 |  | 0.453 | 0.077 | 0.299 | 0.608 | 0.841 | 0.116 | **0.609** | **1.074** |
| 59 | Tb927.4.4220 | Tb927.8.8140 | small GTP-binding rab protein | 0.581 | 0.049 | 0.483 | 0.679 | 0.619 | 0.051 | 0.517 | 0.721 |
| 60 | Tb927.4.4190 | Tb927.8.8150 | C2 calcium/lipid-binding region | 0.353 | 0.041 | 0.271 | 0.436 | 0.245 | 0.034 | 0.177 | 0.314 |
| 61 | Tb927.4.4180 | Tb927.8.8160 |  | 0.692 | 0.057 | 0.578 | 0.806 | 0.730 | 0.058 | 0.615 | 0.846 |
| 62 | Tb927.4.4160 | Tb927.8.8170 | CheY-like domain | 0.355 | 0.020 | 0.315 | 0.395 | 0.358 | 0.020 | 0.318 | 0.398 |
| 63 | Tb927.4.4150 | Tb927.8.8180 |  | 0.267 | 0.015 | 0.236 | 0.297 | 0.266 | 0.015 | 0.236 | 0.297 |
| 64 | Tb927.4.4140 | Tb927.8.8190 |  | 0.324 | 0.034 | 0.256 | 0.392 | 0.326 | 0.034 | 0.259 | 0.394 |
| 65 | Tb927.4.4130 | Tb927.8.8200 | prefoldin domain | 0.272 | 0.015 | 0.242 | 0.303 | 0.262 | 0.015 | 0.232 | 0.292 |
| 66 | Tb927.4.4120 | Tb927.8.8210 |  | 0.381 | 0.037 | 0.307 | 0.455 | 0.381 | 0.037 | 0.307 | 0.455 |
| 67 | Tb927.4.4060 | Tb927.8.8270 | 3'5'-cyclic nucleotide phosphodiesterase | 0.468 | 0.031 | 0.405 | 0.530 | 0.528 | 0.033 | 0.461 | 0.595 |
| 68 | Tb927.4.4040 | Tb927.8.8280 |  | 0.334 | 0.046 | 0.243 | 0.426 | 0.390 | 0.100 | 0.191 | 0.589 |
| 69 | Tb927.4.4020 | Tb927.8.8290 | amino acid transporter AATP5 | 0.272 | 0.021 | 0.230 | 0.314 | 0.250 | 0.020 | 0.210 | 0.291 |
| 70 | Tb927.4.3970 | Tb927.8.8320 |  | 0.552 | 0.055 | 0.443 | 0.662 | 0.792 | 0.071 | 0.650 | 0.934 |
| 71 | Tb927.4.3950 | Tb927.8.8330 | cytoskeleton-associated protein CAP5.5 | 0.396 | 0.022 | 0.352 | 0.440 | 0.376 | 0.021 | 0.334 | 0.419 |
| 72 | Tb927.4.3920 | Tb927.8.8340 | TMH, CRAL-TRIO lipid binding domain | 0.258 | 0.025 | 0.208 | 0.308 | 0.343 | 0.030 | 0.283 | 0.403 |
| 73 | Tb927.4.3910 | Tb927.8.8350 | mitotic centromer-associated kinesin | 0.334 | 0.024 | 0.287 | 0.381 | 0.381 | 0.024 | 0.333 | 0.430 |
| 74 | Tb927.4.3880 | Tb927.8.8360 | receptor-type adenylate cyclase GRESAG 4 | - | - | - | - | - | - | - | - |

Significant asymmetry in substitution rate, i.e., where 95% confidence intervals around genetic distances do not overlap, are shown in bold.
