# Supplementary material for: Evolutionary consequences of a large duplication event in Trypanosoma brucei: Chromosomes 4 and 8 are partial duplicons
Source: BMC Genomics. 2007 Nov 23;8:432. doi: 10.1186/1471-2164-8-432 (PMC2212663; doi:10.1186/1471-2164-8-432)
Supplement: Additional data file 5 — Table S4. Evidence for positive and negative selection per codon for single-copy loci (i.e., duplicate lost) and retained paralogs within the duplicated region. [file 1471-2164-8-432-S5.doc]

Table S4. Evidence for positive and negative selection per codon for single-copy loci (i.e., duplicate lost) and retained paralogs within the duplicated region.

| Locus* | Identifier |  | Selection analysis: | |  |  |  |  |  |  |
| --- | --- | --- | --- | --- | --- | --- | --- | --- | --- | --- |
|  | Chr4 | Chr8 | With outgroup | |  |  |  |  | Without outgroup | |
|  |  |  | Method | Global w | + | - | Pos sites | Neg sites | Pos sites | Neg sites |
|  |  |  |  |  |  |  |  |  |  |  |
| 1 | Tb927.4.5390 | Tb927.8.6930 | SLAC | 0.132 | 0.121 | 0.198 | 0 | 1 |  |  |
|  |  |  | FEL |  |  |  | 0 | 40 |  |  |
|  |  |  | REL |  |  |  | 0 | 0 | 0 | 0 |
| 2 | Tb927.4.5380 | Tb927.8.6940 | SLAC | 0.215 | 0.21 | 0.292 | 0 | 0 |  |  |
|  |  |  | FEL |  |  |  | 0 | 122 |  |  |
|  |  |  | REL |  |  |  | 1 | 0 | 0 | 0 |
| 3 | Tb927.4.5370 | Tb927.8.6950 | SLAC | 0.114 | 0.089 | 0.196 | 0 | 0 |  |  |
|  |  |  | FEL |  |  |  | 0 | 14 |  |  |
|  |  |  | REL |  |  |  | 0 | 0 | 0 | 0 |
| 4 | Tb927.4.5360 | Tb927.8.6960 | SLAC | 0.146 | 0.133 | 0.217 | 0 | 0 |  |  |
|  |  |  | FEL |  |  |  | 0 | 41 |  |  |
|  |  |  | REL |  |  |  | 1 | 0 | 0 | 7 |
| 5 | Tb927.4.5350 | Tb927.8.6970 | SLAC | 0.194 | 0.183 | 0.265 | 0 | 0 |  |  |
|  |  |  | FEL |  |  |  | 0 | 27 |  |  |
|  |  |  | REL |  |  |  | 5 | 0 | 0 | 2 |
| 6 | Tb927.4.5340 | Tb927.8.6980 | SLAC | 0.382 | 0.369 | 0.462 | 0 | 0 |  |  |
|  |  |  | FEL |  |  |  | 0 | 54 |  |  |
|  |  |  | REL |  |  |  | 2 | 65 | 67 | 0 |
|  |  | Tb927.8.7000 | SLAC | 0.303 | 0.269 | 0.446 | 0 | 0 |  |  |
|  |  |  | FEL |  |  |  | 0 | 15 |  |  |
|  |  |  | REL |  |  |  | 0 | 0 |  |  |
|  |  | Tb927.8.7010 | SLAC | 0.199 | 0.176 | 0.295 | 0 | 0 |  |  |
|  |  |  | FEL |  |  |  | 0 | 9 |  |  |
|  |  |  | REL |  |  |  | 12 | 1 |  |  |
|  |  | Tb927.8.7020 | SLAC | 0.175 | 0.169 | 0.244 | 0 | 0 |  |  |
|  |  |  | FEL |  |  |  | 0 | 104 |  |  |
|  |  |  | REL |  |  |  | 0 | 0 |  |  |
|  |  | Tb927.8.7030 | SLAC | 0.283 | 0.26 | 0.393 | 0 | 0 |  |  |
|  |  |  | FEL |  |  |  | 0 | 23 |  |  |
|  |  |  | REL |  |  |  | 0 | 0 |  |  |
|  |  | Tb927.8.7040 | SLAC | 0.181 | 0.156 | 0.274 | 0 | 0 |  |  |
|  |  |  | FEL |  |  |  | 0 | 14 |  |  |
|  |  |  | REL |  |  |  | 0 | 0 |  |  |
|  |  | Tb927.8.7050 | SLAC | 0.456 | 0.429 | 0.6 | 0 | 0 |  |  |
|  |  |  | FEL |  |  |  | 0 | 27 |  |  |
|  |  |  | REL |  |  |  | 0 | 0 |  |  |
| 7 | Tb927.4.5330 | Tb927.8.7060 | SLAC | 0.6 | 0.584 | 0.701 | 0 | 5 |  |  |
|  |  |  | FEL |  |  |  | 1 | 40 |  |  |
|  |  |  | REL |  |  |  | 0 | 9 | 0 | 0 |
|  |  | Tb927.8.7070 | SLAC | 0.325 | 0.302 | 0.438 | 0 | 0 |  |  |
|  |  |  | FEL |  |  |  | 0 | 14 |  |  |
|  |  |  | REL |  |  |  | 0 | 0 |  |  |
|  |  | Tb927.8.7080 | SLAC | 0.32 | 0.314 | 0.418 | 0 | 0 |  |  |
|  |  |  | FEL |  |  |  | 0 | 96 |  |  |
|  |  |  | REL |  |  |  | 0 | 0 |  |  |
| 8 | Tb927.4.5320 | Tb927.8.7090 | SLAC | 0.333 | 0.325 | 0.418 | 0 | 0 |  |  |
|  |  |  | FEL |  |  |  | 0 | 84 |  |  |
|  |  |  | REL |  |  |  | 5 | 340 | 7 | 6 |
|  |  | Tb927.8.7100 | SLAC | 0.135 | 0.133 | 0.19 | 0 | 0 |  |  |
|  |  |  | FEL |  |  |  | 0 | 242 |  |  |
|  |  |  | REL |  |  |  | 0 | 0 |  |  |
| 9 | Tb927.4.5310 | Tb927.8.7110 | SLAC | 0.149 | 0.139 | 0.215 | 0 | 0 |  |  |
|  |  |  | FEL |  |  |  | 0 | 56 |  |  |
|  |  |  | REL |  |  |  | 0 | 0 | 0 | 0 |
|  |  | Tb927.8.7120 | SLAC | 0.192 | 0.18 | 0.275 | 0 | 0 |  |  |
|  |  |  | FEL |  |  |  | 0 | 21 |  |  |
|  |  |  | REL |  |  |  | 1 | 151 |  |  |
|  |  | Tb927.8.7130 | SLAC | 0.316 | 0.283 | 0.439 | 0 | 0 |  |  |
|  |  |  | FEL |  |  |  | 0 | 4 |  |  |
|  |  |  | REL |  |  |  | 0 | 0 |  |  |
| 10 | Tb927.4.5300 | Tb927.8.7140 | SLAC | 0.289 | 0.271 | 0.364 | 0 | 1 |  |  |
|  |  |  | FEL |  |  |  | 1 | 44 |  |  |
|  |  |  | REL |  |  |  | 15 | 192 | 20 | 7 |
|  |  | Tb927.8.7170 | SLAC | 0.225 | 0.209 | 0.313 | 0 | 0 |  |  |
|  |  |  | FEL |  |  |  | 0 | 22 |  |  |
|  |  |  | REL |  |  |  | 0 | 5 |  |  |
| 11 | Tb927.4.5230 | Tb927.8.7180 | SLAC | 0.279 | 0.261 | 0.364 | 0 | 0 |  |  |
|  |  |  | FEL |  |  |  | 0 | 31 |  |  |
|  |  |  | REL |  |  |  | 0 | 44 | 1 | 2 |
| 12 | Tb927.4.5220 | Tb927.8.7190 | SLAC | 0.315 | 0.302 | 0.415 | 0 | 0 |  |  |
|  |  |  | FEL |  |  |  | 0 | 31 |  |  |
|  |  |  | REL |  |  |  | 6 | 77 | 19 | 7 |
|  |  | Tb927.8.7200 | SLAC | 0.179 | 0.174 | 0.243 | 0 | 0 |  |  |
|  |  |  | FEL |  |  |  | 0 | 117 |  |  |
|  |  |  | REL |  |  |  | 0 | 0 |  |  |
|  | Tb927.4.5210 |  | SLAC | No *T. congolense* sequence | | |  |  |  |  |
|  |  |  | FEL |  |  |  |  |  |  |  |
|  |  |  | REL |  |  |  |  |  |  |  |
|  | Tb927.4.5200 |  | SLAC | 0.153 | 0.14 | 0.216 | 0 | 0 |  |  |
|  |  |  | FEL |  |  |  | 0 | 44 |  |  |
|  |  |  | REL |  |  |  | 0 | 12 |  |  |
| 13 | Tb927.4.5190 | Tb927.8.7210 | SLAC | 0.103 | 0.09 | 0.169 | 0 | 0 |  |  |
|  |  |  | FEL |  |  |  | 0 | 46 |  |  |
|  |  |  | REL |  |  |  | 0 | 0 | 0 | 61 |
| 14 | Tb927.4.5180 | Tb927.8.7220 | SLAC | 0.201 | 0.192 | 0.266 | 0 | 0 |  |  |
|  |  |  | FEL |  |  |  | 0 | 73 |  |  |
|  |  |  | REL |  |  |  | 4 | 135 | 2 | 49 |
|  | Tb927.4.5170 |  | SLAC | 0.157 | 0.134 | 0.243 | 0 | 0 |  |  |
|  |  |  | FEL |  |  |  | 0 | 11 |  |  |
|  |  |  | REL |  |  |  | 0 | 0 |  |  |
| 15 | Tb927.4.5160 | Tb927.8.7230 | SLAC | 0.172 | 0.158 | 0.244 | 0 | 1 |  |  |
|  |  |  | FEL |  |  |  | 0 | 54 |  |  |
|  |  |  | REL |  |  |  | 0 | 5 | 0 | 0 |
| 16 | Tb927.4.5150 | Tb927.8.7240 | SLAC | 0.263 | 0.245 | 0.341 | 0 | 1 |  |  |
|  |  |  | FEL |  |  |  | 0 | 35 |  |  |
|  |  |  | REL |  |  |  | 0 | 0 | 0 | 24 |
| 17 | Tb927.4.5140 | Tb927.8.7250 | SLAC | 0.112 | 0.098 | 0.17 | 0 | 0 |  |  |
|  |  |  | FEL |  |  |  | 0 | 49 |  |  |
|  |  |  | REL |  |  |  | 0 | 0 | 0 | 29 |
|  | Tb927.4.5130 |  | SLAC | 0.2 | 0.179 | 0.285 | 0 | 0 |  |  |
|  |  |  | FEL |  |  |  | 0 | 20 |  |  |
|  |  |  | REL |  |  |  | 0 | 11 |  |  |
| 18 | Tb927.4.5120 | Tb927.8.7260 | SLAC | 0.304 | 0.299 | 0.394 | 0 | 0 |  |  |
|  |  |  | FEL |  |  |  | 0 | 152 |  |  |
|  |  |  | REL |  |  |  | 0 | 44 | 0 | 22 |
|  | Tb927.4.5110 |  | SLAC | 0.383 | 0.359 | 0.494 | 0 | 0 |  |  |
|  |  |  | FEL |  |  |  | 0 | 18 |  |  |
|  |  |  | REL |  |  |  | 0 | 0 |  |  |
| 19 | Tb927.4.5100 | Tb927.8.7270 | SLAC | 0.107 | 0.1 | 0.156 | 0 | 0 |  |  |
|  |  |  | FEL |  |  |  | 0 | 110 |  |  |
|  |  |  | REL |  |  |  | 2 | 126 | 0 | 1 |
|  |  | Tb927.8.7280 | SLAC | 0.182 | 0.163 | 0.267 | 0 | 0 |  |  |
|  |  |  | FEL |  |  |  | 0 | 20 |  |  |
|  |  |  | REL |  |  |  | 4 | 5 |  |  |
|  |  | Tb927.8.7290 | SLAC | 0.136 | 0.128 | 0.196 | 0 | 0 |  |  |
|  |  |  | FEL |  |  |  | 0 | 60 |  |  |
|  |  |  | REL |  |  |  | 17 | 0 |  |  |
|  | Tb927.4.5070 |  | SLAC | No *T. congolense* sequence | | |  |  |  |  |
|  |  |  | FEL |  |  |  |  |  |  |  |
|  |  |  | REL |  |  |  |  |  |  |  |
|  | Tb927.4.5060 |  | SLAC | 0.397 | 0.359 | 0.52 | 0 | 0 |  |  |
|  |  |  | FEL |  |  |  | 0 | 13 |  |  |
|  |  |  | REL |  |  |  | 0 | 0 |  |  |
|  |  | Tb927.8.7360 | SLAC | 0.296 | 0.288 | 0.383 | 0 | 0 |  |  |
|  |  |  | FEL |  |  |  | 0 | 81 |  |  |
|  |  |  | REL |  |  |  | 0 | 0 |  |  |
| 20 | Tb927.4.5050 | Tb927.8.7380 | SLAC | 0.079 | 0.072 | 0.119 | 0 | 3 |  |  |
|  |  |  | FEL |  |  |  | 0 | 70 |  |  |
|  |  |  | REL |  |  |  | 0 | 146 | 0 | 9 |
|  | Tb927.4.5040 |  | SLAC | No *T. congolense* sequence | | |  |  |  |  |
|  |  |  | FEL |  |  |  |  |  |  |  |
|  |  |  | REL |  |  |  |  |  |  |  |
| 21 | Tb927.4.5030 | Tb927.8.7390 | SLAC | 0.072 | 0.062 | 0.112 | 0 | 0 |  |  |
|  |  |  | FEL |  |  |  | 0 | 65 |  |  |
|  |  |  | REL |  |  |  | 5 | 286 | 3 | 24 |
| 22 | Tb927.4.5020 | Tb927.8.7400 | SLAC | 0.064 | 0.062 | 0.094 | 0 | 0 |  |  |
|  |  |  | FEL |  |  |  | 0 | 267 |  |  |
|  |  |  | REL |  |  |  | 3 | 28 | 0 | 0 |
| 23 | Tb927.4.5010 | Tb927.8.7410 | SLAC | 0.112 | 0.102 | 0.172 | 0 | 0 |  |  |
|  |  |  | FEL |  |  |  | 0 | 40 |  |  |
|  |  |  | REL |  |  |  | 0 | 0 | 0 | 0 |
| 24 | Tb927.4.5000 | Tb927.8.7420 | SLAC | 0.291 | 0.282 | 0.374 | 0 | 1 |  |  |
|  |  |  | FEL |  |  |  | 0 | 79 |  |  |
|  |  |  | REL |  |  |  | 189 | 0 | 0 | 0 |
| 25 | Tb927.4.4990 | Tb927.8.7430 | SLAC | 0.075 | 0.048 | 0.156 | 0 | 0 |  |  |
|  |  |  | FEL |  |  |  | 0 | 11 |  |  |
|  |  |  | REL |  |  |  | 0 | 0 | 0 | 0 |
|  | Tb927.4.4980 |  | SLAC | 0.161 | 0.137 | 0.247 | 0 | 0 |  |  |
|  |  |  | FEL |  |  |  | 0 | 21 |  |  |
|  |  |  | REL |  |  |  | 0 | 0 |  |  |
|  |  | Tb927.8.7440 | SLAC | 0.309 | 0.286 | 0.398 | 0 | 0 |  |  |
|  |  |  | FEL |  |  |  | 0 | 30 |  |  |
|  |  |  | REL |  |  |  | 0 | 0 |  |  |
| 26 | Tb927.4.4970 | Tb927.8.7450 | SLAC | 0.114 | 0.102 | 0.167 | 0 | 0 |  |  |
|  |  |  | FEL |  |  |  | 0 | 50 |  |  |
|  |  |  | REL |  |  |  | 0 | 0 | 18 | 0 |
| 27 | Tb927.4.4960 | Tb927.8.7460 | SLAC | 0.188 | 0.176 | 0.246 | 0 | 0 |  |  |
|  |  |  | FEL |  |  |  | 0 | 76 |  |  |
|  |  |  | REL |  |  |  | 7 | 289 | 49 | 0 |
| 28 | Tb927.4.4950 | Tb927.8.7470 | SLAC | 0.211 | 0.199 | 0.283 | 0 | 0 |  |  |
|  |  |  | FEL |  |  |  | 0 | 42 |  |  |
|  |  |  | REL |  |  |  | 0 | 5 | 0 | 0 |
| 29 | Tb927.4.4940 | Tb927.8.7480 | SLAC | 0.299 | 0.291 | 0.377 | 0 | 0 |  |  |
|  |  |  | FEL |  |  |  | 0 | 88 |  |  |
|  |  |  | REL |  |  |  | 0 | 85 | 0 | 0 |
| 30 | Tb927.4.4930 | Tb927.8.7490 | SLAC | 0.185 | 0.18 | 0.25 | 0 | 1 |  |  |
|  |  |  | FEL |  |  |  | 0 | 123 |  |  |
|  |  |  | REL |  |  |  | 0 | 0 | 0 | 0 |
| 31 | Tb927.4.4920 | Tb927.8.7500 | SLAC | 0.413 | 0.363 | 0.508 | 0 | 0 |  |  |
|  |  |  | FEL |  |  |  | 0 | 13 |  |  |
|  |  |  | REL |  |  |  | 6 | 63 | 15 | 0 |
|  |  | Tb927.8.7510 | SLAC | 0.167 | 0.153 | 0.237 | 0 | 0 |  |  |
|  |  |  | FEL |  |  |  | 0 | 34 |  |  |
|  |  |  | REL |  |  |  | 0 | 2 |  |  |
|  |  | Tb927.8.7520 | SLAC | 0.224 | 0.214 | 0.3 | 0 | 0 |  |  |
|  |  |  | FEL |  |  |  | 0 | 50 |  |  |
|  |  |  | REL |  |  |  | 5 | 0 |  |  |
| 32 | Tb927.4.4910 | Tb927.8.7530 | SLAC | 0.146 | 0.136 | 0.211 | 0 | 1 |  |  |
|  |  |  | FEL |  |  |  | 0 | 72 |  |  |
|  |  |  | REL |  |  |  | 0 | 43 | 0 | 95 |
| 33 | Tb927.4.4900 | Tb927.8.7550 | SLAC | 0.449 | 0.433 | 0.547 | 0 | 0 |  |  |
|  |  |  | FEL |  |  |  | 0 | 25 |  |  |
|  |  |  | REL |  |  |  | 0 | 18 | 0 | 0 |
| 34 | Tb927.4.4890 | Tb927.8.7560 | SLAC | 0.238 | 0.232 | 0.324 | 0 | 0 |  |  |
|  |  |  | FEL |  |  |  | 0 | 88 |  |  |
|  |  |  | REL |  |  |  | 1 | 122 | 0 | 0 |
|  |  | Tb927.8.7570 | SLAC | 0.146 | 0.137 | 0.209 | 0 | 0 |  |  |
|  |  |  | FEL |  |  |  | 0 | 64 |  |  |
|  |  |  | REL |  |  |  | 0 | 0 |  |  |
| 35 | Tb927.4.4880 | Tb927.8.7580 | SLAC | 0.263 | 0.257 | 0.343 | 0 | 0 |  |  |
|  |  |  | FEL |  |  |  | 1 | 115 |  |  |
|  |  |  | REL |  |  |  | 0 | 205 | 0 | 0 |
| 36 | Tb927.4.4870 | Tb927.8.7600 | SLAC | 0.182 | 0.175 | 0.256 | 0 | 2 |  |  |
|  |  |  | FEL |  |  |  | 1 | 100 |  |  |
|  |  |  | REL |  |  |  | 0 | 252 | 0 | 3 |
| 37 | Tb927.4.4810 | Tb927.8.7710 | SLAC | 0.302 | 0.282 | 0.402 | 0 | 0 |  |  |
|  |  |  | FEL |  |  |  | 0 | 34 |  |  |
|  |  |  | REL |  |  |  | 0 | 50 | 29 | 0 |
|  | Tb927.4.4800 |  | SLAC | No *T. congolense* sequence | | |  |  |  |  |
|  |  |  | FEL |  |  |  |  |  |  |  |
|  |  |  | REL |  |  |  |  |  |  |  |
| 38 | Tb927.4.4790 | Tb927.8.7720 | SLAC | 0.35 | 0.328 | 0.455 | 0 | 0 |  |  |
|  |  |  | FEL |  |  |  | 0 | 25 |  |  |
|  |  |  | REL |  |  |  | 0 | 20 | 0 | 0 |
|  | Tb927.4.4760 |  | SLAC | 0.279 | 0.272 | 0.363 | 0 | 0 |  |  |
|  |  |  | FEL |  |  |  | 0 | 71 |  |  |
|  |  |  | REL |  |  |  | 0 | 0 |  |  |
|  | Tb927.4.4750 |  | SLAC | 0.251 | 0.236 | 0.337 | 0 | 0 |  |  |
|  |  |  | FEL |  |  |  | 0 | 33 |  |  |
|  |  |  | REL |  |  |  | 0 | 0 |  |  |
| 39 | Tb927.4.4740 | Tb927.8.7730 | SLAC | 0.167 | 0.159 | 0.236 | 0 | 0 |  |  |
|  |  |  | FEL |  |  |  | 0 | 97 |  |  |
|  |  |  | REL |  |  |  | 0 | 180 | 0 | 145 |
| 40 | Tb927.4.4730 | Tb927.8.7740 | SLAC | 0.148 | 0.138 | 0.202 | 0 | 1 |  |  |
|  |  |  | FEL |  |  |  | 0 | 45 |  |  |
|  |  |  | REL |  |  |  | 4 | 201 | 40 | 0 |
|  | Tb927.4.4720 |  | SLAC | 0.194 | 0.183 | 0.266 | 0 | 0 |  |  |
|  |  |  | FEL |  |  |  | 0 | 50 |  |  |
|  |  |  | REL |  |  |  | 15 | 0 |  |  |
|  | Tb927.4.4710 |  | SLAC | 0.319 | 0.296 | 0.423 | 0 | 0 |  |  |
|  |  |  | FEL |  |  |  | 0 | 22 |  |  |
|  |  |  | REL |  |  |  | 0 | 0 |  |  |
|  | Tb927.4.4700 |  | SLAC | 0.169 | 0.153 | 0.246 | 0 | 0 |  |  |
|  |  |  | FEL |  |  |  | 0 | 27 |  |  |
|  |  |  | REL |  |  |  | 0 | 0 |  |  |
|  | Tb927.4.4690 |  | SLAC | 0.142 | 0.128 | 0.209 | 0 | 0 |  |  |
|  |  |  | FEL |  |  |  | 0 | 46 |  |  |
|  |  |  | REL |  |  |  | 0 | 0 |  |  |
|  | Tb927.4.4680 |  | SLAC | 0.077 | 0.062 | 0.131 | 0 | 0 |  |  |
|  |  |  | FEL |  |  |  | 0 | 28 |  |  |
|  |  |  | REL |  |  |  | 0 | 45 |  |  |
|  | Tb927.4.4670 |  | SLAC | 0.21 | 0.199 | 0.285 | 0 | 0 |  |  |
|  |  |  | FEL |  |  |  | 0 | 47 |  |  |
|  |  |  | REL |  |  |  | 0 | 0 |  |  |
|  | Tb927.4.4660 |  | SLAC | 0.138 | 0.118 | 0.22 | 0 | 0 |  |  |
|  |  |  | FEL |  |  |  | 0 | 12 |  |  |
|  |  |  | REL |  |  |  | 0 | 0 |  |  |
|  | Tb927.4.4650 |  | SLAC | 0.095 | 0.082 | 0.15 | 0 | 0 |  |  |
|  |  |  | FEL |  |  |  | 0 | 33 |  |  |
|  |  |  | REL |  |  |  | 7 | 3 |  |  |
|  | Tb927.4.4640 |  | SLAC | 0.089 | 0.072 | 0.149 | 0 | 0 |  |  |
|  |  |  | FEL |  |  |  | 0 | 21 |  |  |
|  |  |  | REL |  |  |  | 0 | 0 |  |  |
|  | Tb927.4.4630 |  | SLAC | 0.145 | 0.118 | 0.24 | 0 | 0 |  |  |
|  |  |  | FEL |  |  |  | 0 | 14 |  |  |
|  |  |  | REL |  |  |  | 0 | 0 |  |  |
|  | Tb927.4.4620 |  | SLAC | 0.061 | 0.043 | 0.117 | 0 | 0 |  |  |
|  |  |  | FEL |  |  |  | 0 | 23 |  |  |
|  |  |  | REL |  |  |  | 3 | 0 |  |  |
|  | Tb927.4.4610 |  | SLAC | 0.219 | 0.202 | 0.304 | 0 | 0 |  |  |
|  |  |  | FEL |  |  |  | 0 | 20 |  |  |
|  |  |  | REL |  |  |  | 1 | 3 |  |  |
|  | Tb927.4.4600 |  | SLAC | 0.072 | 0.061 | 0.124 | 0 | 0 |  |  |
|  |  |  | FEL |  |  |  | 0 | 35 |  |  |
|  |  |  | REL |  |  |  | 0 | 127 |  |  |
|  | Tb927.4.4590 |  | SLAC | 0.264 | 0.251 | 0.344 | 0 | 1 |  |  |
|  |  |  | FEL |  |  |  | 0 | 51 |  |  |
|  |  |  | REL |  |  |  | 0 | 0 |  |  |
| 41 | Tb927.4.4580 | Tb927.8.7750 | SLAC | 0.357 | 0.351 | 0.431 | 0 | 0 |  |  |
|  |  |  | FEL |  |  |  | 3 | 120 |  |  |
|  |  |  | REL |  |  |  | 0 | 87 | 0 | 0 |
| 42 | Tb927.4.4570 | Tb927.8.7760 | SLAC | 0.286 | 0.28 | 0.364 | 0 | 2 |  |  |
|  |  |  | FEL |  |  |  | 0 | 143 |  |  |
|  |  |  | REL |  |  |  | 0 | 180 | 0 | 0 |
|  | Tb927.4.4560 |  | SLAC | 0.258 | 0.251 | 0.332 | 0 | 0 |  |  |
|  |  |  | FEL |  |  |  | 0 | 81 |  |  |
|  |  |  | REL |  |  |  | 0 | 0 |  |  |
|  |  | Tb927.8.7770 | SLAC | 0.244 | 0.232 | 0.324 | 0 | 1 |  |  |
|  |  |  | FEL |  |  |  | 1 | 45 |  |  |
|  |  |  | REL |  |  |  | 0 | 0 |  |  |
| 43 | Tb927.4.4550 | Tb927.8.7780 | SLAC | 0.199 | 0.193 | 0.261 | 0 | 1 |  |  |
|  |  |  | FEL |  |  |  | 0 | 158 |  |  |
|  |  |  | REL |  |  |  | 11 | 17 | 5 | 7 |
| 44 | Tb927.4.4540 | Tb927.8.7790 | SLAC | 0.115 | 0.097 | 0.188 | 0 | 0 |  |  |
|  |  |  | FEL |  |  |  | 0 | 37 |  |  |
|  |  |  | REL |  |  |  | 0 | 80 | 0 | 0 |
| 45 | Tb927.4.4530 | Tb927.8.7800 | SLAC | 0.072 | 0.061 | 0.121 | 0 | 0 |  |  |
|  |  |  | FEL |  |  |  | 0 | 35 |  |  |
|  |  |  | REL |  |  |  | 0 | 34 | 0 | 0 |
|  |  | Tb927.8.7810 | SLAC | 0.194 | 0.178 | 0.274 | 0 | 0 |  |  |
|  |  |  | FEL |  |  |  | 0 | 24 |  |  |
|  |  |  | REL |  |  |  | 0 | 0 |  |  |
| 46 | Tb927.4.4520 | Tb927.8.7820 | SLAC | 0.342 | 0.33 | 0.426 | 0 | 0 |  |  |
|  |  |  | FEL |  |  |  | 1 | 57 |  |  |
|  |  |  | REL |  |  |  | 1 | 73 | 6 | 0 |
|  | Tb927.4.4510 |  | SLAC | 0.29 | 0.284 | 0.365 | 0 | 0 |  |  |
|  |  |  | FEL |  |  |  | 0 | 125 |  |  |
|  |  |  | REL |  |  |  | 0 | 0 |  |  |
| 47 | Tb927.4.4500 | Tb927.8.7830 | SLAC | 0.435 | 0.427 | 0.513 | 0 | 0 |  |  |
|  |  |  | FEL |  |  |  | 3 | 83 |  |  |
|  |  |  | REL |  |  |  | 0 | 64 | 0 | 0 |
|  | Tb927.4.4490 |  | SLAC | 0.178 | 0.173 | 0.237 | 0 | 1 |  |  |
|  |  |  | FEL |  |  |  | 0 | 138 |  |  |
|  |  |  | REL |  |  |  | 0 | 0 |  |  |
|  |  | Tb927.8.7840 | SLAC | 0.324 | 0.312 | 0.408 | 0 | 0 |  |  |
|  |  |  | FEL |  |  |  | 0 | 55 |  |  |
|  |  |  | REL |  |  |  | 0 | 0 |  |  |
| 48 | Tb927.4.4480 | Tb927.8.7850 | SLAC | 0.296 | 0.291 | 0.371 | 0 | 2 |  |  |
|  |  |  | FEL |  |  |  | 0 | 134 |  |  |
|  |  |  | REL |  |  |  | 6 | 180 | 0 | 0 |
| 49 | Tb927.4.4470 | Tb927.8.7860 | SLAC | 0.256 | 0.253 | 0.335 | 0 | 2 |  |  |
|  |  |  | FEL |  |  |  | 0 | 214 |  |  |
|  |  |  | REL |  |  |  | 2 | 187 | 20 | 0 |
| 50 | Tb927.4.4400 | Tb927.8.7950 | SLAC | 0.369 | 0.365 | 0.447 | 0 | 10 |  |  |
|  |  |  | FEL |  |  |  | 4 | 191 |  |  |
|  |  |  | REL |  |  |  | 0 | 121 | 2 | 0 |
| 51 | Tb927.4.4380 | Tb927.8.7980 | SLAC | 0.062 | 0.057 | 0.096 | 0 | 0 |  |  |
|  |  |  | FEL |  |  |  | 0 | 106 |  |  |
|  |  |  | REL |  |  |  | 24 | 0 | 0 | 0 |
|  |  | Tb927.8.7990 | SLAC | No *T. congolense* sequence | | |  |  |  |  |
|  |  |  | FEL |  |  |  |  |  |  |  |
|  |  |  | REL |  |  |  |  |  |  |  |
| 52 | Tb927.4.4370 | Tb927.8.8000 | SLAC | 0.216 | 0.207 | 0.28 | 0 | 0 |  |  |
|  |  |  | FEL |  |  |  | 0 | 91 |  |  |
|  |  |  | REL |  |  |  | 49 | 68 | 8 | 0 |
|  |  | Tb927.8.8010 | SLAC | 0.367 | 0.48 | 0.461 | 0 | 1 |  |  |
|  |  |  | FEL |  |  |  | 0 | 33 |  |  |
|  |  |  | REL |  |  |  | 0 | 0 |  |  |
| 53 | Tb927.4.4360 | Tb927.8.8020 | SLAC | 0.111 | 0.103 | 0.168 | 0 | 1 |  |  |
|  |  |  | FEL |  |  |  | 0 | 92 |  |  |
|  |  |  | REL |  |  |  | 0 | 194 | 0 | 0 |
| 54 | Tb927.4.4350 | Tb927.8.8030 | SLAC | 0.253 | 0.236 | 0.351 | 0 | 0 |  |  |
|  |  |  | FEL |  |  |  | 0 | 36 |  |  |
|  |  |  | REL |  |  |  | 0 | 58 | 0 | 0 |
|  | Tb927.4.4340 |  | SLAC | 0.167 | 0.154 | 0.237 | 0 | 0 |  |  |
|  |  |  | FEL |  |  |  | 0 | 40 |  |  |
|  |  |  | REL |  |  |  | 0 | 0 |  |  |
| 55 | Tb927.4.4330 | Tb927.8.8040 | SLAC | 0.141 | 0.128 | 0.204 | 0 | 0 |  |  |
|  |  |  | FEL |  |  |  | 0 | 54 |  |  |
|  |  |  | REL |  |  |  | 0 | 149 | 7 | 0 |
|  | Tb927.4.4320 |  | SLAC | 0.113 | 0.091 | 0.184 | 0 | 0 |  |  |
|  |  |  | FEL |  |  |  | 0 | 12 |  |  |
|  |  |  | REL |  |  |  | 3 | 0 |  |  |
| 56 | Tb927.4.4310 | Tb927.8.8050 | SLAC | 0.402 | 0.39 | 0.492 | 0 | 0 |  |  |
|  |  |  | FEL |  |  |  | 0 | 65 |  |  |
|  |  |  | REL |  |  |  | 0 | 59 | 0 | 0 |
|  | Tb927.4.4300 |  | SLAC | 0.216 | 0.205 | 0.293 | 0 | 0 |  |  |
|  |  |  | FEL |  |  |  | 0 | 55 |  |  |
|  |  |  | REL |  |  |  | 2 | 44 |  |  |
| 57 | Tb927.4.4290 | Tb927.8.8090 | SLAC | 0.208 | 0.199 | 0.291 | 0 | 0 |  |  |
|  |  |  | FEL |  |  |  | 0 | 64 |  |  |
|  |  |  | REL |  |  |  | 7 | 142 | 0 | 0 |
| 58 | Tb927.4.4240 | Tb927.8.8070 | SLAC | 0.26 | 0.233 | 0.368 | 0 | 0 |  |  |
|  |  |  | FEL |  |  |  | 0 | 13 |  |  |
|  |  |  | REL |  |  |  | 1 | 15 | 0 | 0 |
|  | Tb927.4.4230 |  | SLAC | 0.256 | 0.247 | 0.333 | 0 | 0 |  |  |
|  |  |  | FEL |  |  |  | 0 | 79 |  |  |
|  |  |  | REL |  |  |  | 0 | 0 |  |  |
| 59 | Tb927.4.4220 | Tb927.8.8140 | SLAC | 0.378 | 0.367 | 0.465 | 0 | 0 |  |  |
|  |  |  | FEL |  |  |  | 0 | 65 |  |  |
|  |  |  | REL |  |  |  | 0 | 112 | 0 | 0 |
|  | Tb927.4.4210 |  | SLAC | 0.146 | 0.141 | 0.204 | 0 | 0 |  |  |
|  |  |  | FEL |  |  |  | 0 | 106 |  |  |
|  |  |  | REL |  |  |  | 0 | 0 |  |  |
|  | Tb927.4.4200 |  | SLAC | 0.227 | 0.203 | 0.312 | 0 | 0 |  |  |
|  |  |  | FEL |  |  |  | 0 | 20 |  |  |
|  |  |  | REL |  |  |  | 0 | 3 |  |  |
| 60 | Tb927.4.4190 | Tb927.8.8150 | SLAC | 0.142 | 0.128 | 0.216 | 0 | 0 |  |  |
|  |  |  | FEL |  |  |  | 0 | 41 |  |  |
|  |  |  | REL |  |  |  | 0 | 76 | 1 | 4 |
| 61 | Tb927.4.4180 | Tb927.8.8160 | SLAC | 0.324 | 0.317 | 0.404 | 0 | 0 |  |  |
|  |  |  | FEL |  |  |  | 0 | 87 |  |  |
|  |  |  | REL |  |  |  | 0 | 61 | 0 | 1 |
|  | Tb927.4.4170 |  | SLAC | 0.257 | 0.233 | 0.344 | 0 | 0 |  |  |
|  |  |  | FEL |  |  |  | 0 | 24 |  |  |
|  |  |  | REL |  |  |  | 0 | 0 |  |  |
| 62 | Tb927.4.4160 | Tb927.8.8170 | SLAC | 0.282 | 0.273 | 0.345 | 0 | 1 |  |  |
|  |  |  | FEL |  |  |  | 1 | 94 |  |  |
|  |  |  | REL |  |  |  | 3 | 181 | 5 | 0 |
| 63 | Tb927.4.4150 | Tb927.8.8180 | SLAC | 0.155 | 0.149 | 0.213 | 0 | 0 |  |  |
|  |  |  | FEL |  |  |  | 0 | 105 |  |  |
|  |  |  | REL |  |  |  | 0 | 0 | 0 | 0 |
| 64 | Tb927.4.4140 | Tb927.8.8190 | SLAC | 0.299 | 0.286 | 0.382 | 0 | 0 |  |  |
|  |  |  | FEL |  |  |  | 0 | 51 |  |  |
|  |  |  | REL |  |  |  | 0 | 0 | 0 | 0 |
| 65 | Tb927.4.4130 | Tb927.8.8200 | SLAC | 0.117 | 0.112 | 0.165 | 0 | 7 |  |  |
|  |  |  | FEL |  |  |  | 0 | 116 |  |  |
|  |  |  | REL |  |  |  | 0 | 0 | 24 | 54 |
| 66 | Tb927.4.4120 | Tb927.8.8210 | SLAC | 0.088 | 0.07 | 0.157 | 0 | 0 |  |  |
|  |  |  | FEL |  |  |  | 0 | 12 |  |  |
|  |  |  | REL |  |  |  | 0 | 33 | 0 | 0 |
|  | Tb927.4.4110 |  | SLAC | 0.138 | 0.129 | 0.2 | 0 | 0 |  |  |
|  |  |  | FEL |  |  |  | 0 | 47 |  |  |
|  |  |  | REL |  |  |  | 0 | 0 |  |  |
|  | Tb927.4.4100 |  | SLAC | 0.314 | 0.298 | 0.397 | 0 | 0 |  |  |
|  |  |  | FEL |  |  |  | 0 | 36 |  |  |
|  |  |  | REL |  |  |  | 0 | 2 |  |  |
|  | Tb927.4.4090 |  | SLAC | 0.245 | 0.227 | 0.337 | 0 | 0 |  |  |
|  |  |  | FEL |  |  |  | 0 | 28 |  |  |
|  |  |  | REL |  |  |  | 0 | 3 |  |  |
|  | Tb927.4.4080 |  | SLAC | 0.094 | 0.083 | 0.149 | 0 | 0 |  |  |
|  |  |  | FEL |  |  |  | 0 | 47 |  |  |
|  |  |  | REL |  |  |  | 0 | 0 |  |  |
|  | Tb927.4.4070 |  | SLAC | 0.113 | 0.102 | 0.17 | 0 | 0 |  |  |
|  |  |  | FEL |  |  |  | 0 | 52 |  |  |
|  |  |  | REL |  |  |  | 0 | 167 |  |  |
| 67 | Tb927.4.4060 | Tb927.8.8270 | SLAC | 0.311 | 0.305 | 0.393 | 0 | 2 |  |  |
|  |  |  | FEL |  |  |  | 1 | 127 |  |  |
|  |  |  | REL |  |  |  | 0 | 107 | 0 | 0 |
|  | Tb927.4.4050 |  | SLAC | 0.157 | 0.148 | 0.221 | 0 | 0 |  |  |
|  |  |  | FEL |  |  |  | 0 | 64 |  |  |
|  |  |  | REL |  |  |  | 0 | 0 |  |  |
| 68 | Tb927.4.4040 | Tb927.8.8280 | SLAC | 0.317 | 0.3 | 0.421 | 0 | 0 |  |  |
|  |  |  | FEL |  |  |  | 0 | 24 |  |  |
|  |  |  | REL |  |  |  | 0 | 0 | 0 | 0 |
|  | Tb927.4.4030 |  | SLAC | 0.197 | 0.187 | 0.264 | 0 | 0 |  |  |
|  |  |  | FEL |  |  |  | 0 | 75 |  |  |
|  |  |  | REL |  |  |  | 1 | 0 |  |  |
| 69 | Tb927.4.4020 | Tb927.8.8290 | SLAC | 0.175 | 0.169 | 0.245 | 0 | 0 |  |  |
|  |  |  | FEL |  |  |  | 0 | 105 |  |  |
|  |  |  | REL |  |  |  | 15 | 224 | 0 | 6 |
| 70 | Tb927.4.3970 | Tb927.8.8320 | SLAC | 0.367 | 0.347 | 0.464 | 0 | 0 |  |  |
|  |  |  | FEL |  |  |  | 0 | 22 |  |  |
|  |  |  | REL |  |  |  | 3 | 50 | 0 | 0 |
|  | Tb927.4.3960 |  | SLAC | 0.199 | 0.179 | 0.295 | 0 | 1 |  |  |
|  |  |  | FEL |  |  |  | 0 | 27 |  |  |
|  |  |  | REL |  |  |  | 0 | 0 |  |  |
| 71 | Tb927.4.3950 | Tb927.8.8330 | SLAC | 0.249 | 0.242 | 0.318 | 0 | 0 |  |  |
|  |  |  | FEL |  |  |  | 1 | 115 |  |  |
|  |  |  | REL |  |  |  | 14 | 458 | 27 | 41 |
|  | Tb927.4.3940 |  | SLAC | 0.161 | 0.154 | 0.219 | 0 | 0 |  |  |
|  |  |  | FEL |  |  |  | 0 | 98 |  |  |
|  |  |  | REL |  |  |  | 2 | 0 |  |  |
|  | Tb927.4.3930 |  | SLAC | 0.106 | 0.097 | 0.158 | 0 | 0 |  |  |
|  |  |  | FEL |  |  |  | 0 | 76 |  |  |
|  |  |  | REL |  |  |  | 0 | 0 |  |  |
| 72 | Tb927.4.3920 | Tb927.8.8340 | SLAC | 0.131 | 0.122 | 0.196 | 0 | 0 |  |  |
|  |  |  | FEL |  |  |  | 0 | 69 |  |  |
|  |  |  | REL |  |  |  | 0 | 142 | 0 | 11 |
| 73 | Tb927.4.3910 | Tb927.8.8350 | SLAC | 0.213 | 0.207 | 0.272 | 0 | 0 |  |  |
|  |  |  | FEL |  |  |  | 0 | 135 |  |  |
|  |  |  | REL |  |  |  | 0 | 413 | 0 | 0 |
|  | Tb927.4.3900 |  | SLAC | 0.151 | 0.14 | 0.22 | 0 | 0 |  |  |
|  |  |  | FEL |  |  |  | 0 | 40 |  |  |
|  |  |  | REL |  |  |  | 0 | 13 |  |  |
|  | Tb927.4.3890 |  | SLAC | 0.126 | 0.121 | 0.178 | 0 | 1 |  |  |
|  |  |  | FEL |  |  |  | 0 | 117 |  |  |
|  |  |  | REL |  |  |  | 0 | 0 |  |  |
| 74 | Tb927.4.3880 | Tb927.8.8360 | SLAC | 0.238 | 0.231 | 0.326 | 0 | 0 |  |  |
|  |  |  | FEL |  |  |  | 0 | 74 |  |  |
|  |  |  | REL |  |  |  | 0 | 143 | 0 | 0 |
