# Supplementary material for: Evolutionary consequences of a large duplication event in Trypanosoma brucei: Chromosomes 4 and 8 are partial duplicons
Source: BMC Genomics. 2007 Nov 23;8:432. doi: 10.1186/1471-2164-8-432 (PMC2212663; doi:10.1186/1471-2164-8-432)
Supplement: Additional data file 6 — Table S5. Comparisons of ratios of invariable to variable mutations at synonymous (i.e., silent, S) and non-synonymous (i.e., replacement, R) sites, between shared paralogs. [file 1471-2164-8-432-S6.doc]

Table S5. Comparisons of ratios of invariable to variable mutations at synonymous (i.e., silent, S) and non-synonymous (i.e., replacement, R) sites, between shared paralogs.

| **Locus** | **Identifier** |  | **Description** | **Mutation type:** | |  |  | **G statistic** | **P** | **Ratio _R_** | **Ratio _S_** | **Ratio _RS_** |
| --- | --- | --- | --- | --- | --- | --- | --- | --- | --- | --- | --- | --- |
|  | Chr4 | Chr8 |  | RI | RV | SI | SV |  |  |  |  |  |
|  |  |  |  |  |  |  |  |  |  |  |  |  |
| 1 | Tb927.4.5390 | Tb927.8.6930 | serine/threonine-protein kinase NrkA | 87 | 13 | 124 | 13 | 0.707193 | 0.500000 > P > 0.200000 | 6.6923 | 9.5385 | 0.7016 |
| 2 | Tb927.4.5380 | Tb927.8.6940 | alcohol dehydrogenase-like | **436** | **10** | **310** | **21** | **8.155326** | **0.005000 > P > 0.000000** | **43.6** | **14.762** | **2.9535** |
| 3 | Tb927.4.5370 | Tb927.8.6950 | dynein light chain 2B | 27 | 1 | 35 | 2 | 0.106502 | 0.900000 > P > 0.500000 | 27 | 17.5 | 1.5429 |
| 4 | Tb927.4.5360 | Tb927.8.6960 | TMH/SP | 100 | 9 | 107 | 7 | 0.363805 | 0.900000 > P > 0.500000 | 11.111 | 15.286 | 0.7269 |
| 5 | Tb927.4.5350 | Tb927.8.6970 | 3-methylcrotonyl-CoA carboxylase | 191 | 23 | 175 | 13 | 1.806818 | 0.200000 > P > 0.100000 | 8.3043 | 13.462 | 0.6169 |
| 6 | Tb927.4.5340 | Tb927.8.6980 |  | **356** | **284** | **147** | **191** | **13.03674** | **0.005000 > P > 0.000000** | **1.2535** | **0.7696** | **1.6287** |
| 7 | Tb927.4.5330 | Tb927.8.7060 |  | - | - | - | - | - | - | - | - | - |
| 8 | Tb927.4.5320 | Tb927.8.7090 |  | **197** | **384** | **105** | **314** | **9.130732** | **0.005000 > P > 0.000000** | **0.513** | **0.3344** | **1.5342** |
| 9 | Tb927.4.5310 | Tb927.8.7110 | serine/threonine-protein kinase A | 53 | 98 | 74 | 141 | 0.017979 | 0.900000 > P > 0.500000 | 0.5408 | 0.5248 | 1.0305 |
| 10 | Tb927.4.5300 | Tb927.8.7140 | UDP-GlcNAc-dependent glycosyltransferase | 38 | 194 | 29 | 183 | 0.627987 | 0.500000 > P > 0.200000 | 0.1959 | 0.1585 | 1.236 |
| 11 | Tb927.4.5230 | Tb927.8.7180 |  | 19 | 303 | 13 | 132 | 1.382611 | 0.500000 > P > 0.200000 | 0.0627 | 0.0985 | 0.6367 |
| 12 | Tb927.4.5220 | Tb927.8.7190 |  | 56 | 293 | 21 | 173 | 2.872694 | 0.100000 > P > 0.050000 | 0.1911 | 0.1214 | 1.5745 |
| 13 | Tb927.4.5190 | Tb927.8.7210 |  | 10 | 44 | 13 | 71 | 0.211965 | 0.900000 > P > 0.500000 | 0.2273 | 0.1831 | 1.2413 |
| 14 | Tb927.4.5180 | Tb927.8.7220 | protein kinase | 87 | 51 | 140 | 60 | 1.772287 | 0.200000 > P > 0.100000 | 1.7059 | 2.3333 | 0.7311 |
| 15 | Tb927.4.5160 | Tb927.8.7230 | TMH/SP | 62 | 75 | 51 | 89 | 2.224449 | 0.200000 > P > 0.100000 | 0.8267 | 0.573 | 1.4426 |
| 16 | Tb927.4.5150 | Tb927.8.7240 |  | 89 | 54 | 67 | 37 | 0.122931 | 0.900000 > P > 0.500000 | 1.6481 | 1.8108 | 0.9102 |
| 17 | Tb927.4.5140 | Tb927.8.7250 |  | 37 | 15 | 58 | 31 | 0.532553 | 0.500000 > P > 0.200000 | 2.4667 | 1.871 | 1.3184 |
| 18 | Tb927.4.5120 | Tb927.8.7260 | kinetoplast-associated protein | 172 | 1171 | 75 | 675 | 3.716079 | 0.100000 > P > 0.050000 | 0.1469 | 0.1111 | 1.3219 |
| 19 | Tb927.4.5100 | Tb927.8.7270 | TMH/SP | 68 | 110 | 64 | 148 | 2.759098 | 0.100000 > P > 0.050000 | 0.6182 | 0.4324 | 1.4295 |
| 20 | Tb927.4.5050 | Tb927.8.7380 | dihydrolipoamide dehydrogenase | 111 | 21 | 153 | 15 | 3.336505 | 0.100000 > P > 0.050000 | 5.2857 | 10.2 | 0.5182 |
| 21 | Tb927.4.5030 | Tb927.8.7390 | serine/threonine protein phosphatase PP1 | 45 | 21 | 91 | 36 | 0.247082 | 0.900000 > P > 0.500000 | 2.1429 | 2.5278 | 0.8477 |
| 22 | Tb927.4.5020 | Tb927.8.7400 | RNA polymerase IIA largest subunit | 48 | 1 | 286 | 14 | 0.781803 | 0.500000 > P > 0.200000 | 48 | 20.429 | 2.3497 |
| 23 | Tb927.4.5010 | Tb927.8.7410 | calreticulin | 111 | 7 | 86 | 2 | 1.644346 | 0.200000 > P > 0.100000 | 15.857 | 43 | 0.3688 |
| 24 | Tb927.4.5000 | Tb927.8.7420 | C2 calcium/lipid-binding region | **256** | **5** | **210** | **15** | **6.937288** | **0.005000 > P > 0.000000** | **51.2** | **14** | **3.6571** |
| 25 | Tb927.4.4990 | Tb927.8.7430 | ubiquinol-cytochrome C reductase hinge protein | 10 | 1 | 15 | 1 | 0.059736 | 0.900000 > P > 0.500000 | 10 | 15 | 0.6667 |
| 26 | Tb927.4.4970 | Tb927.8.7450 | myosin heavy chain kinase A | 83 | 27 | 74 | 38 | 2.348638 | 0.200000 > P > 0.100000 | 3.0741 | 1.9474 | 1.5786 |
| 27 | Tb927.4.4960 | Tb927.8.7460 | metal-ion transporter | 108 | 95 | 95 | 87 | 0.038423 | 0.900000 > P > 0.500000 | 1.1368 | 1.092 | 1.0411 |
| 28 | Tb927.4.4950 | Tb927.8.7470 | aldehyde dehydrogenase, WD40 repeat | 186 | 8 | 129 | 5 | 0.030978 | 0.900000 > P > 0.500000 | 23.25 | 25.8 | 0.9012 |
| 29 | Tb927.4.4940 | Tb927.8.7480 | Phosphopantetheine attachment site | **135** | **484** | **53** | **317** | **8.673923** | **0.005000 > P > 0.000000** | **0.2789** | **0.1672** | **1.6683** |
| 30 | Tb927.4.4930 | Tb927.8.7490 |  | 333 | 20 | 359 | 24 | 0.116828 | 0.900000 > P > 0.500000 | 16.65 | 14.958 | 1.1131 |
| 31 | Tb927.4.4920 | Tb927.8.7500 | TMH/SP | 5 | 214 | 2 | 115 | 0.117878 | 0.900000 > P > 0.500000 | 0.0234 | 0.0174 | 1.3435 |
| 32 | Tb927.4.4910 | Tb927.8.7530 | 3,2-trans-enoyl-CoA isomerase | 49 | 118 | 56 | 134 | 0.000729 | 0.990000 > P > 0.950000 | 0.4153 | 0.4179 | 0.9936 |
| 33 | Tb927.4.4900 | Tb927.8.7550 |  | 120 | 639 | 49 | 273 | 0.061288 | 0.900000 > P > 0.500000 | 0.1878 | 0.1795 | 1.0463 |
| 34 | Tb927.4.4890 | Tb927.8.7560 | TMH | 83 | 326 | 47 | 284 | 4.740567 | 0.050000 > P > 0.025000 | 0.2546 | 0.1655 | 1.5384 |
| 35 | Tb927.4.4880 | Tb927.8.7580 | TMH/SP, Zinc finger, C3HC4 type | **201** | **539** | **75** | **400** | **22.050253** | **0.005000 > P > 0.000000** | **0.3729** | **0.1875** | **1.9889** |
| 36 | Tb927.4.4870 | Tb927.8.7600 | amino acid transporter | **132** | **279** | **89** | **379** | **19.927397** | **0.005000 > P > 0.000000** | **0.4731** | **0.2348** | **2.0147** |
| 37 | Tb927.4.4810 | Tb927.8.7710 | TMH | **54** | **191** | **41** | **287** | **9.081055** | **0.005000 > P > 0.000000** | **0.2827** | **0.1429** | **1.9791** |
| 38 | Tb927.4.4790 | Tb927.8.7720 | TMH/SP | 52 | 223 | 19 | 142 | 3.882606 | 0.050000 > P > 0.025000 | 0.2332 | 0.1338 | 1.7427 |
| 39 | Tb927.4.4740 | Tb927.8.7730 | longevity-assurance protein | 31 | 229 | 18 | 137 | 0.008691 | 0.950000 > P > 0.900000 | 0.1354 | 0.1314 | 1.0303 |
| 40 | Tb927.4.4730 | Tb927.8.7740 | amino acid transporter | **139** | **60** | **83** | **77** | **12.094011** | **0.005000 > P > 0.000000** | **2.3167** | **1.0779** | **2.1492** |
| 41 | Tb927.4.4580 | Tb927.8.7750 | protein kinase | 87 | 429 | 43 | 277 | 1.785358 | 0.200000 > P > 0.100000 | 0.2028 | 0.1552 | 1.3064 |
| 42 | Tb927.4.4570 | Tb927.8.7760 |  | 197 | 743 | 110 | 472 | 0.94747 | 0.500000 > P > 0.200000 | 0.2651 | 0.2331 | 1.1377 |
| 43 | Tb927.4.4550 | Tb927.8.7780 | GPI anchor | 133 | 276 | 115 | 287 | 1.459551 | 0.500000 > P > 0.200000 | 0.4819 | 0.4007 | 1.2026 |
| 44 | Tb927.4.4540 | Tb927.8.7790 | LSD1 zinc finger | 24 | 43 | 18 | 60 | 2.80611 | 0.100000 > P > 0.050000 | 0.5581 | 0.3 | 1.8605 |
| 45 | Tb927.4.4530 | Tb927.8.7800 |  | 146 | 937 | 82 | 597 | 0.734565 | 0.500000 > P > 0.200000 | 0.1558 | 0.1374 | 1.1344 |
| 46 | Tb927.4.4520 | Tb927.8.7820 | cold-shock protein, DNA-binding | - | - | - | - | - | - | - | - | - |
| 47 | Tb927.4.4500 | Tb927.8.7830 |  | 227 | 1338 | 85 | 703 | 6.481813 | 0.025000 > P > 0.010000 | 0.1697 | 0.1209 | 1.4032 |
| 48 | Tb927.4.4480 | Tb927.8.7850 |  | **210** | **598** | **84** | **429** | **17.253714** | **0.005000 > P > 0.000000** | **0.3512** | **0.1958** | **1.7935** |
| 49 | Tb927.4.4470 | Tb927.8.7860 | adenylate cyclase GRESAG 4 | **73** | **502** | **98** | **414** | **8.457828** | **0.005000 > P > 0.000000** | **0.1454** | **0.2367** | **0.6143** |
| 50 | Tb927.4.4400 | Tb927.8.7950 |  | **430** | **1671** | **178** | **905** | **7.648678** | **0.005000 > P > 0.000000** | **0.2573** | **0.1967** | **1.3083** |
| 51 | Tb927.4.4380 | Tb927.8.7980 | V-type H(+)-translocating pyrophosphatase | 126 | 8 | 210 | 14 | 0.011196 | 0.950000 > P > 0.900000 | 15.75 | 15 | 1.05 |
| 52 | Tb927.4.4370 | Tb927.8.8000 |  | 96 | 260 | 95 | 178 | 4.449389 | 0.050000 > P > 0.025000 | 0.3692 | 0.5337 | 0.6918 |
| 53 | Tb927.4.4360 | Tb927.8.8020 | monoglyceride lipase | 51 | 100 | 46 | 158 | 5.434479 | 0.025000 > P > 0.010000 | 0.51 | 0.2911 | 1.7517 |
| 54 | Tb927.4.4350 | Tb927.8.8030 | TMH/SP | 35 | 174 | 18 | 116 | 0.689398 | 0.500000 > P > 0.200000 | 0.2011 | 0.1552 | 1.2963 |
| 55 | Tb927.4.4330 | Tb927.8.8040 | diadenosine tetraphosphatase | 38 | 86 | 47 | 101 | 0.038557 | 0.900000 > P > 0.500000 | 0.4419 | 0.4653 | 0.9495 |
| 56 | Tb927.4.4310 | Tb927.8.8050 | spectrin repeat | - | - | - | - | - | - | - | - | - |
| 57 | Tb927.4.4290 | Tb927.8.8090 | UDP-GlcNAc-dependent glycosyltransferase | **67** | **318** | **24** | **247** | **10.125817** | **0.005000 > P > 0.000000** | **0.2107** | **0.0972** | **2.1684** |
| 58 | Tb927.4.4240 | Tb927.8.8070 |  | 24 | 72 | 5 | 49 | 5.901838 | 0.025000 > P > 0.010000 | 0.3333 | 0.102 | 3.2667 |
| 59 | Tb927.4.4220 | Tb927.8.8140 | small GTP-binding rab protein | - | - | - | - | - | - | - | - | - |
| 60 | Tb927.4.4190 | Tb927.8.8150 | C2 calcium/lipid-binding region | 23 | 64 | 27 | 90 | 0.300131 | 0.900000 > P > 0.500000 | 0.3594 | 0.3 | 1.1979 |
| 61 | Tb927.4.4180 | Tb927.8.8160 |  | **234** | **775** | **83** | **475** | **15.948885** | **0.005000 > P > 0.000000** | **0.3019** | **0.1747** | **1.7279** |
| 62 | Tb927.4.4160 | Tb927.8.8170 | CheY-like domain | 248 | 298 | 190 | 192 | 1.676849 | 0.200000 > P > 0.100000 | 0.8322 | 0.9896 | 0.841 |
| 63 | Tb927.4.4150 | Tb927.8.8180 |  | 277 | 11 | 262 | 13 | 0.277409 | 0.900000 > P > 0.500000 | 25.182 | 20.154 | 1.2495 |
| 64 | Tb927.4.4140 | Tb927.8.8190 |  | 91 | 8 | 67 | 3 | 0.970962 | 0.500000 > P > 0.200000 | 11.375 | 22.333 | 0.5093 |
| 65 | Tb927.4.4130 | Tb927.8.8200 | prefoldin domain | 220 | 127 | 201 | 102 | 0.609732 | 0.500000 > P > 0.200000 | 1.7323 | 1.9706 | 0.8791 |
| 66 | Tb927.4.4120 | Tb927.8.8210 |  | - | - | - | - | - | - | - | - | - |
| 67 | Tb927.4.4060 | Tb927.8.8270 | 3'5'-cyclic nucleotide phosphodiesterase | **263** | **748** | **97** | **485** | **19.050886** | **0.005000 > P > 0.000000** | **0.3516** | **0.2** | **1.758** |
| 68 | Tb927.4.4040 | Tb927.8.8280 |  | 35 | 200 | 19 | 126 | 0.235627 | 0.900000 > P > 0.500000 | 0.175 | 0.1508 | 1.1605 |
| 69 | Tb927.4.4020 | Tb927.8.8290 | amino acid transporter AATP5 | 40 | 184 | 52 | 206 | 0.40831 | 0.900000 > P > 0.500000 | 0.2174 | 0.2524 | 0.8612 |
| 70 | Tb927.4.3970 | Tb927.8.8320 |  | 84 | 541 | 26 | 258 | 3.504682 | 0.100000 > P > 0.050000 | 0.1553 | 0.1008 | 1.5407 |
| 71 | Tb927.4.3950 | Tb927.8.8330 | cytoskeleton-associated protein CAP5.5 | 218 | 185 | 141 | 160 | 3.620479 | 0.100000 > P > 0.050000 | 1.1784 | 0.8813 | 1.3372 |
| 72 | Tb927.4.3920 | Tb927.8.8340 | TMH, CRAL-TRIO lipid binding domain | 31 | 135 | 26 | 151 | 0.972861 | 0.500000 > P > 0.200000 | 0.2296 | 0.1722 | 1.3336 |
| 73 | Tb927.4.3910 | Tb927.8.8350 | mitotic centromer-associated kinesin | 129 | 311 | 126 | 249 | 1.719747 | 0.200000 > P > 0.100000 | 0.4148 | 0.506 | 0.8197 |
| 74 | Tb927.4.3880 | Tb927.8.8360 | receptor-type adenylate cyclase GRESAG 4 | - | - | - | - | - | - | - | - | - |

Comparisons with significant disparity between ratio _R_ and ratio _S_ , to the P < 0.005 level, are shown in bold.
